# Fluoride-activated photothermal system for promoting bacteria-infected wound healing

**DOI:** 10.1186/s12951-023-02091-y

**Published:** 2023-09-15

**Authors:** Yuanchun Du, Zekai Liu, Qingxin Yang, Deshuai Zhen, Yu Liu, Guangfu Feng

**Affiliations:** 1 College of Bioscience and Biotechnology, Hunan Agricultural University, Changsha, 410128 People’s Republic of China; 2 State Key Laboratory of Chemo/Biosensing and Chemometrics, College of Chemistry and Chemical Engineering, Hunan University, Changsha, 410082 People’s Republic of China; 3 College of Public Health, Hengyang Medical School, University of South China, Hengyang, 421001 People’s Republic of China

**Keywords:** Fluoride ions, Photothermal system, Photothermal antibacterial, Wound healing

## Abstract

Although photothermal therapy (PTT) employing nanozymes has shown excellent antibacterial potential, excessive heating generally harms host cells and hinders recovery. Herein, we report an innovative technique for acquiring the programmed temperature by managing the catalytic activity of nanozymes. The photothermal system of CeO_2_ + F^−^ + TMB can obtain precise photothermal temperature by adjusting the concentration of fluoride ions under near-infrared irradiation. At the optimized photothermal temperature, the photothermal system affords fine photothermal antibacterial treatment with high-efficiency antibacterial effects against Staphylococcus aureus and Escherichia coli in vitro. In vivo wound healing experiments confirm that the system can effectively promote fibroblast proliferation, angiogenesis and collagen deposition with remarkable wound healing efficiency. This strategy offers a novel design concept for creating a new generation of PTT and opens the way for the creation of alternative antibiotics.

**Supplementary Information:**

The online version contains supplementary material available at 10.1186/s12951-023-02091-y.

## Introduction

Nanozymes are artificial enzyme mimics with excellent enzyme-like capabilities and distinctive nanomaterial physicochemical characteristics [1]. They offer substantial benefits over natural enzymes, including cheap cost, high stability, outstanding durability, tunable activity, simple storage, and excellent reusability [2]. Following the first report on the peroxidase-like activity of ferromagnetic nanoparticles in 2007, numerous nanozymes based on metals, metal oxides, carbons, metal–organic frameworks (MOFs), covalent organic frameworks (COFs), and other composite nanomaterials have been devised and manufactured [3–7]. Great efforts have also been undertaken to explore the potential of nanozymes in the domains of biosensing, catalysis, clinical medicine, and environmental protection [8–10].

Bacterial infection is a global concern to public health that not only causes several terrible illnesses but also worsens the state of cancer patients and patients with epidemic diseases such as COVID-19 [11, 12]. Antibiotics are now the most widely used and effective method to treat bacterial illness [13]. However, with the overuse of antibiotics, bacterial resistance continues to emerge and spread at an alarming rate [14, 15]. Compared with typical antibiotic treatment, physical stimulation-based antibacterial techniques offer comprehensive and effective antibacterial effects against both common and multidrug-resistant bacteria and may reduce the risk of bacterial resistance [16–18]. Among these physical stimulation options, PTT is an efficient physical sterilizing approach that kills bacteria by inducing protein (including enzyme) denaturation, cell membrane rupture, cell cavitation, and cell fluid evaporation at elevated temperatures [19, 20]. Notably, near-infrared (NIR, 700–1100 nm) laser-mediated PTT affords an unique feature of deep tissue penetration without considerable light-induced cytotoxicity, which is beneficial for establishing antibacterial platforms for various deep therapies [21–23].

In treating infectious illnesses, nanozyme-based NIR PTT has drawn much interest lately owing to its higher efficacy and fewer side responses [24–26]. PTT has specific requirements for photothermal temperature. Excessive temperatures can cause damage to host cells, damaging normal tissues while prolonging wound healing time [27, 28]. When the temperature is too low, the photothermal effect is not apparent, and it is impossible to eradicate microorganisms effectively. For the healing of infected wounds, studies have shown that a suitable temperature (more than 50 °C) efficiently prevents the multiplication of germs [29, 30]. The current photothermal temperature of nanozymes primarily adjusts the temperature by varying the material concentration, the power of the laser irradiation lamp source, and the irradiation time, but it suffers from cumbersome steps and an inability to achieve programmed temperature. Consequently, it is of utmost importance to discover a new strategy to manage the photothermal activity of nanozymes to obtain optimized photothermal temperature for better therapeutic effects.

**Scheme 1:**
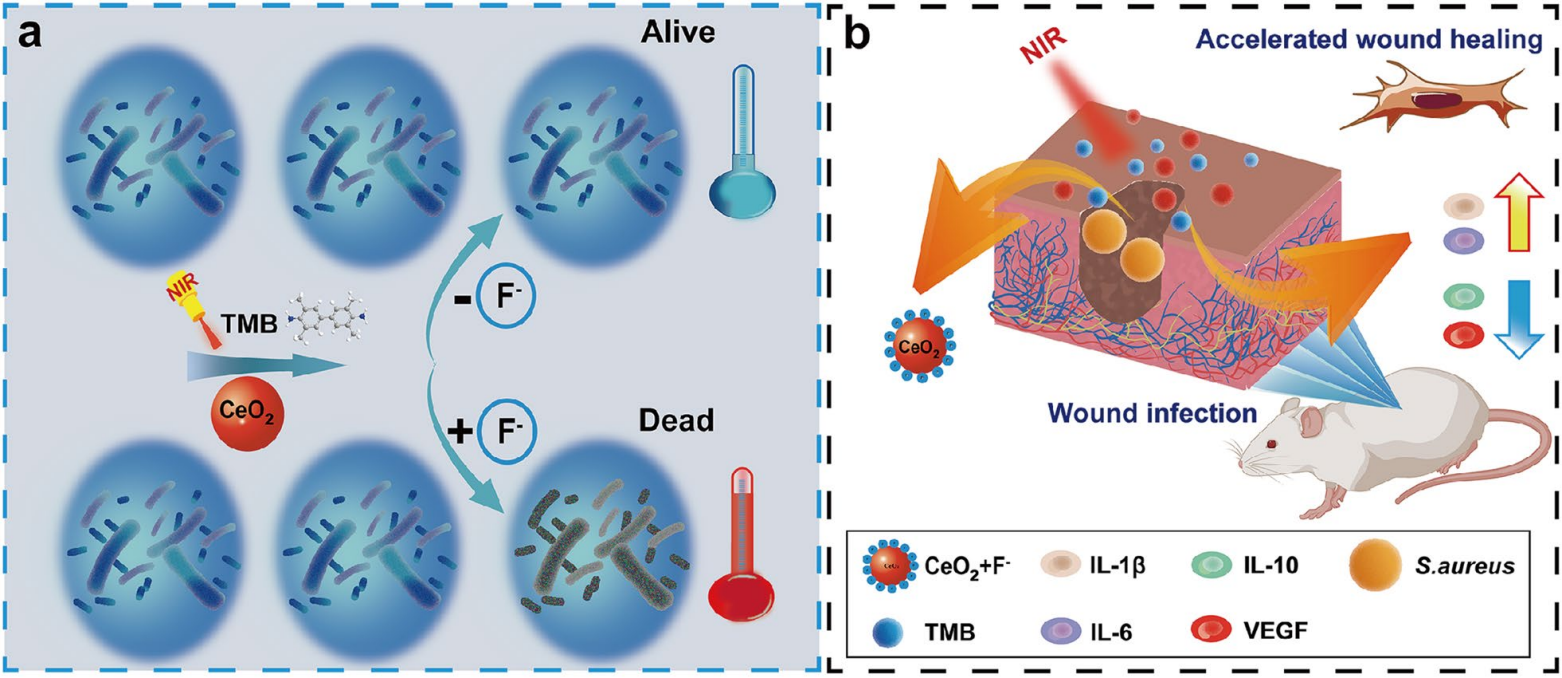
**a** Diagram of photothermal antibacterial for CeO_2_ + TMB with the influence of fluoride ions. **b** Schematic illustrating the CeO_2_ + F^−^ + TMB therapeutic platform for wound healing

Cerium oxide (CeO_2_), an important rare earth metal oxide, contains mixed valence states of CeO_3_^+^ and CeO_4_^+^, high mechanical strength, high conduction band energy, exhibiting powerful catalytic oxidation ability, and offers the benefit of biocompatibility with relatively low or no toxicity to mammalian cells and advantage of being readily biodegradable [31, 32]. CeO_2_ nanoparticles (CeO_2_ NPs) have gained popularity as a nanozyme due to their biocompatibility and multienzyme-like properties [33]. For instance, CeO_2_ NPs display oxidase-like properties without needing hazardous H_2_O_2_ as a co-substrate [34]. The oxidase activity of naked CeO_2_ nanozyme is relatively weak, which limits its use in antibacterial applications to some extent. Recently reports found that fluoride ions (F^−^) could significantly improve the catalytic activity of CeO_2_ nanozyme by altering the surface charge of CeO_2_ for tuning the substrate adsorption affinity and facilitating electron transfer between substrates, oxygen and CeO_2_ [35–37]. Meanwhile, significant advancements have been achieved in the field of photothermal sensing and photothermal treatment based on the principle of substantial photothermal effect generated by the transmutation of TMB into oxidized TMB (oxTMB) [38–43]. Inspired by this, we create a nanozyme-based NIR photothermal system (CeO_2_ + F^−^ + TMB) that can be activated by fluoride ions. The photothermal system can adjust the concentration of oxTMB by adjusting the concentration of fluoride ions, thereby achieving the precise photothermal temperature. In the absence of fluoride ions, the oxidase activity of the bare CeO_2_ nanozyme is feeble, and the conversion of TMB to oxTMB is minimal, resulting in a minor photothermal effect. When fluoride ions are introduced to the system, CeO_2_ oxidase activity steadily increases, and the concentration of fluoride ions is proportional to the concentration of oxTMB produced by TMB oxidation with CeO_2_ nanozyme. Consequently, the photothermal temperature of the CeO_2_ + F^−^ + TMB system is linearly related to the concentration of fluoride ions within a given range. Above this, the temperature of the photothermal system can be changed by altering the concentration of fluoride ions, which can be used for photothermal antibacterial and in vivo wound healing. By altering the concentration of fluoride ions to acquire optimized photothermal temperature, the NIR photothermal system of CeO_2_ + F^−^ + TMB can provide high-efficiency antibacterial effects in vitro against Staphylococcus aureus (*S. aureus*, 99.8%) and Escherichia coli (*E. coli,* 99.3%). Further results show that the photothermal system exhibits good bactericidal activity and biocompatibility in vivo wound infection model (Scheme 1). This work not only sheds new light on the PTT strategy for antibacterial therapy and wound healing but also opens a new avenue for the development of alternative antibiotics.

## Results and discussion

We first performed structural characterization of synthesized CeO_2_ nanoparticles (CeO_2_ NPs). The shape of CeO_2_ NPs was not uniform, and most of those observed in TEM images were granular (Additional file 1: Figure S1). CeO_2_ nanostructures were highly crystalline, as evidenced by HR-TEM images showing a continuous lattice and sharp fringes. In addition, SAED patterns and continuous lattices confirmed the crystalline characteristics and fluorite structure of CeO_2_ NPs. The O and Ce elements were uniformly distributed on the surface of CeO_2_ NPs according to the HAADF-STEM analysis (Additional file 1: Figure S2). XPS spectrum showed the presence of Ce, O and C as the main components and the Ce showed the mixed oxidation state of Ce^3+^ and Ce^4+^, which was very important for the simulated oxidase-like properties of CeO_2_ NPs (Additional file 1: Figure S3). Meantime, the characteristic diffraction peaks of the XRD spectrum at 2θ indicated the remarkable crystalline purity of the CeO_2_ NPs produced (Additional file 1: Figure S4).

**Fig. 1:**
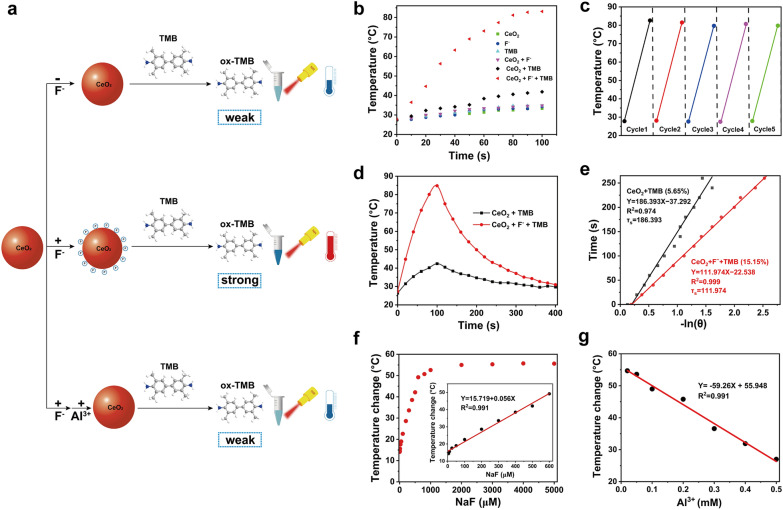
**a** Schematic illustrations of the influence of fluoride ions on the photothermal activity of CeO_2_ + TMB reaction. **b** Photothermal heating curves of CeO_2_, F^−^, TMB, CeO_2_ + F^−^, CeO_2_ + TMB, and CeO_2_ + F^−^ + TMB under 808 nm NIR laser irradiation at 2.3 W/cm^2^. Concentration: CeO_2_ (30 µg/mL), F^−^ (5 mM), TMB (1 mM), acetate buffer (pH 4, 20 mM). **c** Heating and cooling cycles of CeO_2_ + F^−^ + TMB system (CeO_2_: 30 µg/mL, F^−^: 5 mM, TMB: 1 mM) under on–off cycles. **d** Photothermal effects, and **e** corresponding curve fitting of cooling time versus the negative natural logarithm of the driving force temperature during the cooling phase of CeO_2_ + TMB (CeO_2_: 30 µg/mL, TMB: 1 mM) with and without fluoride ions. **f** The linear calibration plots of temperature readings against the various fluoride ions concentrations (0–5000 µM). **g** Relationship between temperature change values from the NIR laser irradiation (808 nm, 2.3 W/cm^2^) under different circumstances of Al^3+^ concentration

We then sought to determine whether fluoride ions could activate the photothermal activity of CeO_2_ + TMB (Fig. 1a). As depicted in Additional file 1: Figure S5, since CeO_2_ and TMB had essentially no absorption in the NIR region, we observed a minor temperature increase of 5.7 °C and 7.4 °C following NIR irradiation (808 nm) for approximately 100 s. A similar temperature rise of 7.4 °C was recorded in the presence of CeO_2_ + F^−^. Due to the low oxidase catalytic activity of the CeO_2_ nanozyme, only a tiny amount of TMB was catalyzed to be oxidized to oxTMB, resulting in weak absorption in the NIR region. The temperature increase of CeO_2_ + TMB under NIR irradiation was only 14.6 °C. As expected, the presence of F^−^ significantly increased the activity of CeO_2_ oxidase to oxidize TMB to oxTMB with strong absorption in the NIR region. Therefore, the CeO_2_ + F^−^ + TMB system showed a robust photothermal effect with a temperature increase of 55.3 °C under NIR irradiation (Fig. 1b). Due to the good photothermal stability of oxTMB, no obvious photobleaching was detected in the CeO_2_ + F^−^ + TMB system after five cycles of on/off NIR irradiation (Fig. 1c). Further, the photothermal conversion efficiency (η) of CeO_2_ + TMB and CeO_2_ + F^−^ + TMB was compared. The η of CeO_2_ + TMB and CeO_2_ + F^−^ + TMB was determined to be 5.65% and 15.15%, validating our speculation that treating naked CeO_2_ NPs with fluoride ions facilitated the improvement of the photothermal performance of CeO_2_ + TMB (Fig. 1d, e). Accordingly, the above findings indicated that F^−^ could effectively stimulate the photothermal conversion efficiency of the CeO_2_ + TMB.

To optimize the performance of the CeO_2_ + F^−^ + TMB system, the photothermal assay investigated the impact of several parameters, including the concentration of CeO_2_ NPs, buffer solution, and light power intensity. The optimized conditions were determined as CeO_2_ NPs concentrate of 30 μg/mL, pH of 4.0, and power intensity of 2.3 W/cm^2^ (Additional file 1: Figure S6). Furthermore, we validated the role of each module in the CeO_2_ + F^−^ + TMB system. In response to the glutathione (GSH), the oxidase activity was wholly eliminated owing to the reduction of CeO_2_ NPs** (**Additional file 1: Figure S7), and the CeO_2_ + F^−^ + TMB system recorded a notable change of photothermal effect with the temperature change value from 56.2 °C to 7.7 °C (Additional file 1: Figure S8). Similarly, with the addition of 8-hydroxyquinoline (8-HQ, blocking the oxidation of TMB) to the CeO_2_ + F^−^ + TMB system, the temperature change value from 56.2 °C to 7.8 °C was observed, verifying the photothermal transformation mediated by oxTMB. Al^3+^, a chelating agent for F^−^, prevents F^−^ from adhering to the surface of CeO_2_ NPs. As predicted, a temperature change value from 56.2 °C to 12.8 °C was seen by the CeO_2_ + F^−^ + TMB system with the addition of Al^3+^ (Additional file 1: Figure S9), which further proved the F^−^ could effectively activate the photothermal conversion efficiency of the CeO_2_ + F^−^ + TMB system.

Based on the above results, we further investigated whether the NIR photothermal system of CeO_2_ + F^−^ + TMB could achieve programmed temperature by adjusting the concentration of F^−^. As shown in Fig. 1f, adding F^−^ at concentrations ranging from 1 μM to 3 mM elicited a rapid dynamic response from the temperature output. The temperature rose linearly with F^−^ concentration from 1 μM to 600 μM. On the contrary, the temperature change values dropped linearly with Al^3+^ concentration from 0.02 mM to 0.5 mM (Fig. 1g**)**. The potential interferences of Cl^−^, Br^−^, I^−^, CO_3_^2−^, H_2_PO_4_^−^, NO_3_^−^, and CH_3_COO^−^ were explored to assess further the specific identification capacity of the CeO_2_ + F^−^ + TMB system for F^−^. As demonstrated in Additional file 1: Figure S10, the addition of potential interferences had no appreciable effect on the temperature change relative to the F^−^, demonstrating the outstanding selectivity of the CeO_2_ + F^−^ + TMB system toward F^−^. Therefore, herein, we constructed a NIR photothermal system of CeO_2_ + F^−^ + TMB that could obtain programmed temperature by varying the concentration of F^−^.

**Fig. 2:**
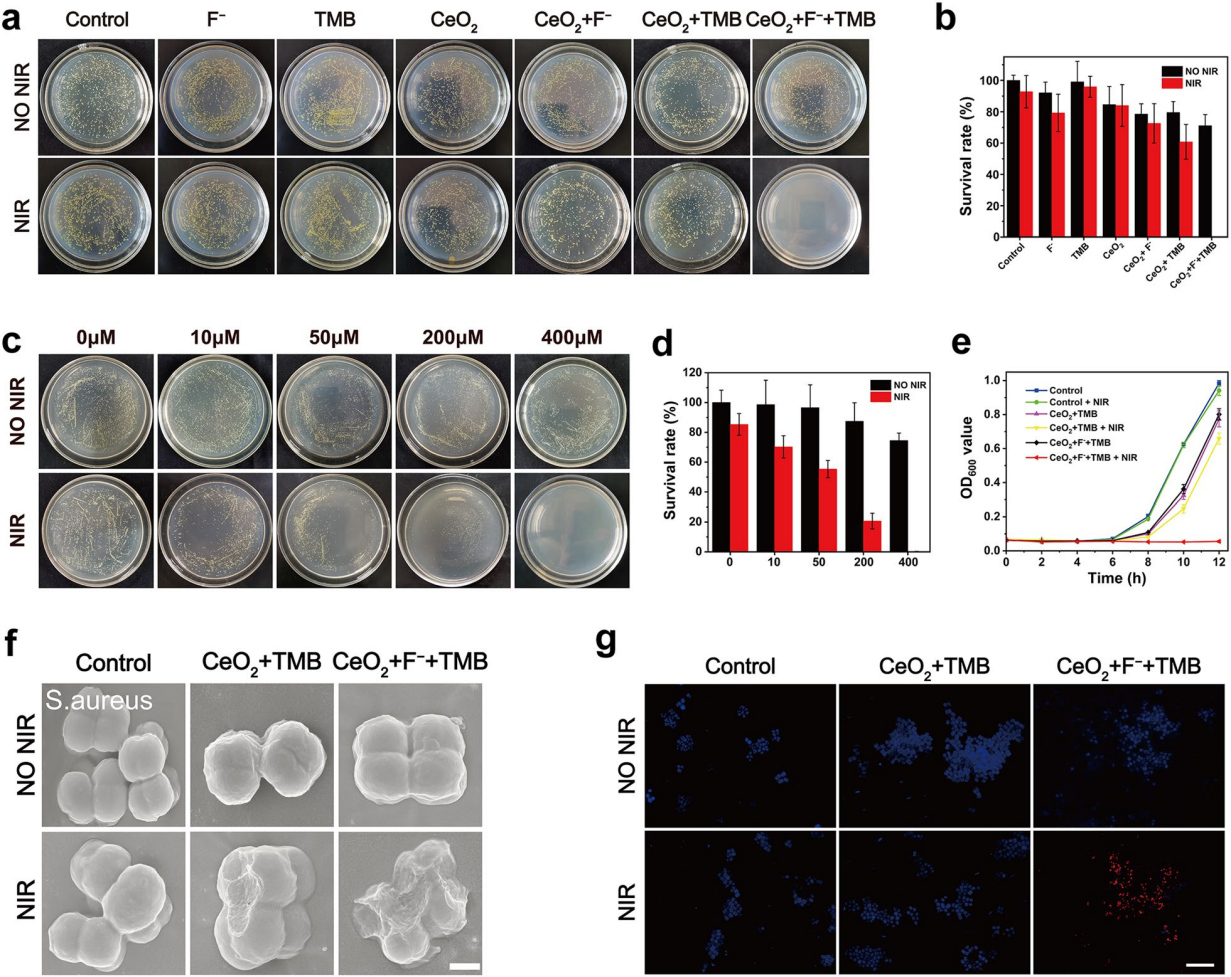
**a** Photographs of bacterial colonies formed by *S. aureus* after exposure to acetate buffer (Control), F^−^, TMB, CeO_2_, CeO_2_ + F^−^, CeO_2_ + TMB and CeO_2_ + F^−^ + TMB without/with NIR irradiation. Concentration: CeO_2_ (30 µg/mL), F^−^ (400 µM), TMB (1 mM), acetate buffer (pH 4, 20 mM). **b** The statistical analysis of survival rates of *S. aureus* exposed to different samples without and with NIR irradiation. **c** Colony images of CeO_2_ + TMB (CeO_2_: 30 µg/mL, TMB: 1 mM) treated *S. aureus* with different F^−^ concentrations (0, 10 µM, 50 µM, 200 µM, 400 µM) under dark and NIR light conditions. **d** The statistical analysis of survival rates of *S. aureus* exposed to CeO_2_ + TMB with varying F^−^ concentrations without and with NIR irradiation. **e** The OD600 value of the supernatant as a function with the time after being treated with various conditions with *S. aureus*. **f** SEM (Scale bar: 500 nm), and **g** Fluorescence images of *S. aureus* samples after treatments with Control (acetate buffer), CeO_2_ + TMB, CeO_2_ + F^−^ + TMB without/with NIR irradiation (Scale bar: 20 μm). Concentration: CeO_2_ (30 µg/mL), F^−^ (400 µM), TMB (1 mM), acetate buffer (pH 4, 20 mM). Error bars represent the standard deviation from the mean (n = 3)

Encouraged by the above data, to this end, we evaluated the antibacterial activity of CeO_2_ + F^−^ + TMB system for PTT in vitro. *S. aureus* (gram-positive organism) and *E. coli* (gram-negative creature) were chosen as typical strains to serve as model bacteria. No statistically significant distinction in the number of colonies was observed between the NIR illumination and dark settings of the control groups for both *S. aureus* and *E. coli* (Fig. 2a and Additional file 1: Figure S11a), proving that NIR illumination alone had no impact on microorganisms. In dark conditions, for the CeO_2_ and TMB group, the survival rates of *S. aureus* (Fig. 2b) were 84.6% and 99.1%, and the survival rates of *E. coli* (Additional file 1: Figure S11b) were close to the control group. Due to the poor photothermal efficiency of CeO_2_ and TMB, the CeO_2_ and TMB group exhibited negligible antibacterial activity when exposed to 808 nm NIR illumination for about 7 min, with survival rates of 83.9% and 95.9% for *S. aureus*, and 101.6% and 81.2% for *E. coli*, respectively. This occurrence proved that CeO_2_ and TMB did not display apparent antibacterial activity under dark and NIR light settings regarding the number of *S. aureus* and *E. coli* colonies. In the CeO_2_ + TMB group, no substantial antibacterial effect was observed for *S. aureus* and *E. coli* in dark conditions. Under NIR irradiation (2.3 W/cm^2^, 7 min), the CeO_2_ + TMB group displayed a poor antibacterial effect with a 60.8% survival rate of *S. aureus* and 63.0% survival rate of *E. coli*, attributing to a low photothermal effect of CeO_2_ + TMB. The *S. aureus* and *E. coli* in the F^−^ groups exhibited survival rates of 79.3% and 89.9% when exposed to 808 nm light, which indicated that the F^−^ barely impacted the bacteria activity. In contrast, under identical conditions for NIR irradiation, the antibacterial rates of the CeO_2_ + F^−^ + TMB group substantially boosted to 99.8% (*S. aureus*) and 99.3% (*E. coli*) in comparison to the CeO_2_ + TMB group, showing that the F^−^ could significantly enhance the photothermal conversion efficiency of the CeO_2_ + TMB for PTT. To find out the association between F^−^ dosage and bacteria sterilization efficiency, the F^−^ concentration was altered to 0, 10, 50, 200, and 400 μM. Notably, under NIR illumination, the antibacterial activity of the CeO_2_ + TMB group against *S. aureus* increased gradually with increasing F^−^ concentration (Fig. 2c). For following antibacterial and wound healing studies, the optimal concentration of F^−^ for PTT was established to be 400 μM due to the ultra-high antibacterial effectiveness (99.7%) and optimal photothermal temperature to guarantee good safety (minimal invasion) and efficient bacterial breakdown (Fig. 2d). Similarly, the antibacterial effect of CeO_2_ + TMB against *E. coli* gradually increased with the increase of F^−^ concentration under NIR irradiation (Additional file 1: Figure S12a), and the optimal antibacterial concentration of F^−^ was also 400 μM (Additional file 1: Figure S12b). The negligible OD600 values of *S. aureus* (Fig. 2e) and *E. coli* (Additional file 1: Figure S13) treated in the CeO_2_ + F^−^ + TMB group under NIR irradiation, similar to the agar plate results, further revealed the potent antibacterial activity of CeO_2_ + TMB could be effectively boosted by F^−^.

To examine the mechanism of antimicrobial action, bacterial SEM testing and bacterial live-dead fluorescence staining under light and dark settings were performed. As shown in Fig. 2f and Additional file 1: Figure S14, for control groups under the NIR illumination and dark settings, *S. aureus* and *E. coli* exhibited typical spherical and rod morphologies with smooth surfaces and undamaged cell walls, respectively similar to those of native living bacteria. After treatment with CeO_2_ + TMB + NIR, a few disturbances could be seen on the cell wall, indicating minimal damage to the integrity of cell walls. However, the treatment of CeO_2_ + F^−^ + TMB + NIR group induced varying degrees of damage to the morphology of the bacterial cells, resulting in compression as well as a roughening of the bacterial surface. The effectiveness of the antibacterial treatment was further evaluated using live/dead fluorescence assays. The living bacteria were stained with blue dye during these tests, while the dead bacteria were stained with red dye. As evidenced by the absence of red fluorescence in Fig. 2g and Additional file 1: Figure S15, most *S. aureus* and *E. coli* were alive under NIR illumination and dark conditions for the control groups. Only a few red luminous cells were found for bacteria treated with CeO_2_ + TMB + NIR group, implying a partial sterilization effect. Notably, treatment with CeO_2_ + F^−^ + TMB + NIR group caused a significant increase in the number of dead cells, as seen by the strong red fluorescence signal, indicating a substantial improvement in antibacterial effectiveness. In short, the potent antibacterial activity of CeO_2_ + TMB could be well activated by F^−^ under NIR illumination.

After thoroughly confirming the outstanding antimicrobial efficiency of CeO_2_ + F^−^ + TMB + NIR in vitro, we further investigated the potential in disinfecting and treating animal wounds. It was vital to assess the biosafety of antibacterial wound healing agents. Before constructing an infectious model in vivo, the cytotoxicity and hemocompatibility of CeO_2_ + F^−^ + TMB were evaluated. We employed NIH3T3 fibroblasts derived from mouse embryos to assess the cytotoxicity. According to the results of the cell counting kit-8 (CCK-8) experiment, CeO_2_ + F^−^ + TMB displayed little cytotoxicity over the 24 h observation period (Additional file 1: Figure S16). To further assess its biocompatibility, the hemolytic performance of CeO_2_ + F^−^ + TMB was measured after it had been grown with mouse red blood cells (RBCs). Positive and negative control RBCs were distributed separately in deionized water and normal saline to evaluate the hemolysis rate. The supernatant (water) from the positive control group had a deep red color (Additional file 1: Figure S17), whereas the supernatant (normal saline) from the negative control group and all other groups was transparent. Less than five percent hemolysis was identified in the group treated with CeO_2_ + F^−^ + TMB, indicating excellent blood compatibility. Together, the CeO_2_ + F^−^ + TMB system with excellent biocompatibility was feasible for in vivo anti-infective treatment.

**Fig. 3:**
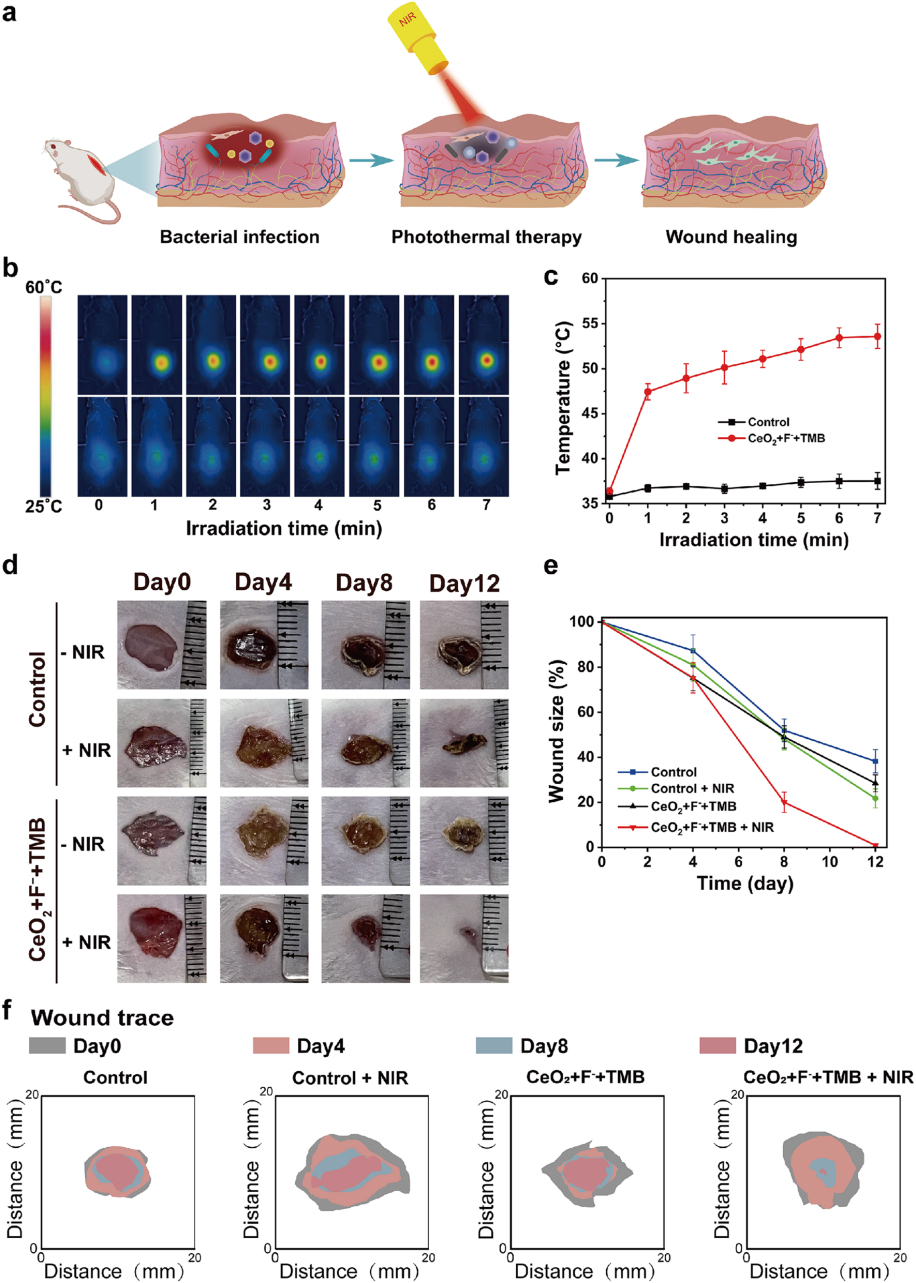
**a** Schematic representations of wound infection model and treatment strategy based on CeO_2_ + F^−^ + TMB therapeutic platform. **b** Infrared thermal pictures, and **c** the rise in temperature at the wound sites of mice at the designated periods under 808 nm NIR illumination (2.3 W cm^2^) for control (acetate buffer (pH 4, 20 mM)).and CeO_2_ + F^−^ + TMB (CeO_2_: 30 µg/mL, F^−^: 400 µM, TMB: 1 mM) groups. **d** Pictures depicting the progression of wound contraction from day 0 to day 12 in response to various treatments. **e** Quantitative analysis of the wound size in each group after different treatments. **f** The dynamic process diagram of wound trace for each treatment within 12 days. Error bars represent the standard deviation from the mean (n = 3)

Inspired by the remarkable antibacterial efficacy and excellent biocompatibility of the CeO_2_ + F^−^ + TMB system, we undertook wound management experiments in vivo. To determine the disinfects abscesses of the CeO_2_ + F^−^ + TMB system, we first established a skin model by injecting 100 μL of *S. aureus* (1 × 10^8^ CFU/mL) into the epidermis of a Balb/c mouse. The phototherapy mechanism for wound treatment was shown in Fig. 3a. We proceed to employ the CeO_2_ + F^−^ + TMB system as a positive control and acetate buffer as a negative control for in vivo wound healing investigations, which divided the mice into four groups, including control, control + NIR, CeO_2_ + F^−^ + TMB, and CeO_2_ + F^−^ + TMB + NIR. The wound receiving NIR phototherapy was monitored using a thermal imaging camera (Fig. 3b). After administering CeO_2_ + F^−^ + TMB injections to mice, the infected area showed a significant photothermal response when exposed to NIR light (808 nm, 2.3 W/cm^2^). The temperature rose rapidly and remained stable at 53.6 °C after about 7 min (Fig. 3c). The photothermal temperature of 53.6 °C in the infected site guaranteed good safety (minimal invasion) and efficient bacterial breakdown. In contrast, there was just a minor temperature change in the control group following acetate buffer treatment under the same conditions. Photographs of wound healing were obtained every four days across different treatment groups (Fig. 3d). The calculation of wound contraction was depicted in Fig. 3e. In all tested groups, the size of the wound decreased over time. Typically, among the four groups, the CeO_2_ + F^−^ + TMB + NIR group displayed, on average, the quickest wound healing (Fig. 3f). Meanwhile, the in vivo photothermal response and wound healing investigations of the CeO_2_ + TMB + NIR group were conducted to further demonstrate the role of F^−^ in CeO_2_ + F^−^ + TMB + NIR photothermal system. As shown in Additional file 1: Figure S18, the temperature slowly increased over time and remained stable at 39.5 °C after about 7 min. The wound size reduced the wound area to 72.2% after 12 days, which was far lower than the 99.1% reduction in wound area by treatemnt of the CeO_2_ + F^−^ + TMB + NIR group (Additional file 1: Figure S19). Therefore, introducing F^−^ could significantly enhance the wound healing effect of the CeO_2_ + TMB + NIR in vivo. For evaluating the sterilization efficiency, bacteria from wound tissue on 2 h and day 7, were cultivated overnight on LB agar plates (Additional file 1: Figure S20). On day 7, the bacteria in the CeO_2_ + F^−^ + TMB + NIR group reduced significantly compared to the other groups. Moreover, to further evaluate the effect of wound healing, we selected the common antibiotic penicillin G as a comparison. The results in Additional file 1: Figure S21 showed that the wound size of thepenicillin G treated group and CeO_2_ + F^−^ + TMB + NIR treated group reduced the wound area to 94.1% and 99.1% after 12 days, respectively. Judging from the above data, the CeO_2_ + F^−^ + TMB NIR photothermal system could substantially improve bacteria-infected wound healing.

**Fig. 4:**
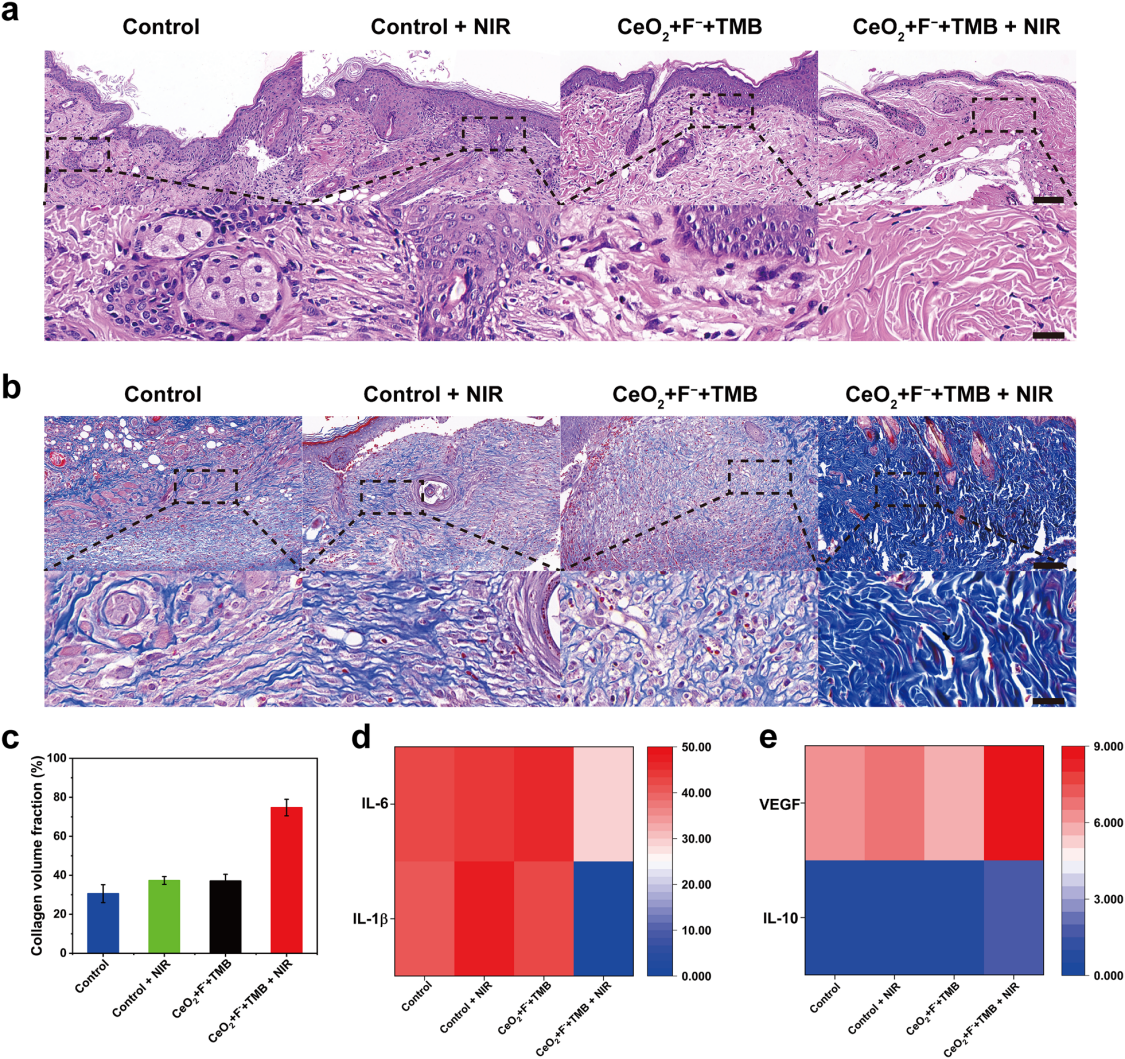
**a** H&E (Scale bar: the top of 100 µm and the bottom of 25 µm), and **b** Masson staining of wound tissues treated with Control, Control + NIR, CeO_2_ + F^−^ + TMB, and CeO_2_ + F^−^ + TMB + NIR (Scale bar: the top of 100 µm and the bottom of 25 µm). Concentration: CeO_2_ (30 µg/mL), F^−^ (400 µM), TMB (1 mM), acetate buffer (pH 4, 20 mM). **c** Quantification of collagen volume fraction. **d** Levels of IL-1β and IL-6 (pg/mg), **e** IL-10 and VEGF (pg/mg). Error bars represent the standard deviation from the mean (n = 3)

Furthermore, alteration in skin tissue was evaluated to corroborate the detail of the aforementioned wound healing. The histological investigation was performed using the H&E (hematoxylin and eosin) staining and Masson’s trichrome staining. At a certain period after treatment, mice were euthanized, and tissues were obtained from the control, control + NIR, CeO_2_ + F^−^ + TMB, and CeO_2_ + F^−^ + TMB + NIR groups. Results from H&E staining revealed that on day 8, on the wound treated with CeO_2_ + F^−^ + TMB + NIR group, the epidermal layer was intact, and there were fewer inflammatory cells (blood cells and neutrophils), whereas the epidermal layer was compromised, and there were more inflammatory cells emerged in the skin tissue of control, control + NIR, and CeO_2_ + F^−^ + TMB groups (Fig. 4a). In addition, abundant fibroblasts and blood vessels were seen in CeO_2_ + F^−^ + TMB + NIR group, indicating improved tissue regeneration and wound healing. Masson's trichrome staining revealed that on day 8, the wound treated with CeO_2_ + F^−^ + TMB + NIR group had well-established collagen fibers and a dermal layer. In contrast, the wounds in the other treatment groups included many collagen fibers that had not been repaired (Fig. 4b). Additionally, compared to other groups, scar width treated with the CeO_2_ + F^−^ + TMB + NIR group decreased rapidly (Additional file 1: Figure S22). Furthermore, more collagen fibers were observed in the CeO_2_ + F^−^ + TMB + NIR group than in other treatment groups **(**Fig. 4c). Numerous inflammatory cytokines are essential in the initiation and progression of inflammatory disease. As demonstrated in Fig. 4d, e, the levels of pro-inflammatory cytokines IL-1β and IL-6 in the CeO_2_ + F^−^ + TMB + NIR group were substantially lower than in the other treatment groups, while the levels of anti-inflammatory cytokines IL-10 and growth factors VEGF were significantly higher. The data on inflammatory cytokines revealed that the treatment of the CeO_2_ + F^−^ + TMB + NIR group could effectively decrease the expression of IL-1β and IL-6 while up-regulating the expression of IL-10 and VEGF, hence reducing the inflammatory response. In short, wounds treated with CeO_2_ + F^−^ + TMB + NIR demonstrated minimal inflammation, good hemostasis, a high collagen fiber content, thick granulation tissue and quick wound healing. Consequently, CeO_2_ + F^−^ + TMB + NIR could accelerate the phase transition from hemostasis to inflammation, proliferation, and remodeling, facilitating wound healing.

**Fig. 5:**
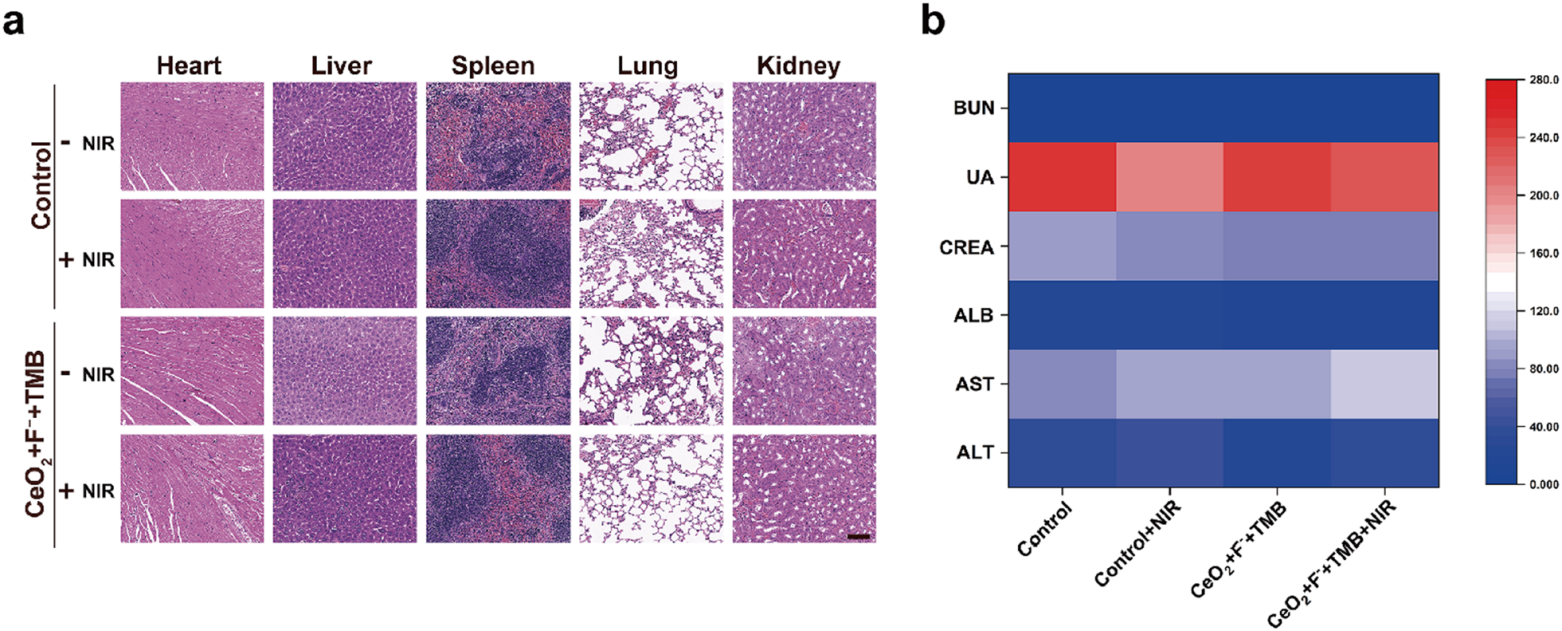
**a** Mice infected with *S. aureus* and subjected to various treatments were sliced and stained with H&E. Scale bar: 100 µm. **b** Blood biochemistry analysis

Meanwhile, the in vivo biosafety of our proposed CeO_2_ + F^−^ + TMB + NIR antimicrobial system was further investigated to demonstrate its potential for future bacterial suppression applications. Monitoring the mice's body weight throughout the experimental treatments indicated no significant weight loss in either the CeO_2_ + F^−^ + TMB + NIR group or other treatment groups, proving that these treatments were not systemically toxic to mice (Additional file 1: Figure S23). Besides, the primary organs of the treated mice were taken for H&E staining, including the heart, liver, spleen, lung, and kidney, and no abnormalities or lesions were seen on the biopsy photographs (Fig. 5a). Additionally, the routine blood tests and blood biochemical analyses of the mice in the CeO_2_ + F^−^ + TMB + NIR group and other treatment groups showed no significant differences in any of the parameters, proving that the kidney and liver functions were unharmed (Fig. 5b). Collectively, these findings repeatedly validated the good biological security of antimicrobial therapy utilizing the CeO_2_ + F^−^ + TMB NIR photothermal system, revealing the great potential in clinical translation.

## Conclusion

In summary, we have built a unique NIR photothermal system of CeO_2_ + F^−^ + TMB that can acquire the programmed temperature by managing the photothermal activity of nanozymes. By adjusting the concentration of fluoride ions to achieve optimized photothermal temperature, the CeO_2_ + F^−^ + TMB NIR photothermal system not only could realize high-efficiency antibacterial effects against *S. aureus* and *E. coli* in vitro, but also could guarantee good safety of minimal invasion. The CeO_2_ + F^−^ + TMB NIR photothermal system could also substantially improve bacteria-infected wound healing via anti-inflammatory by reduction of pro-inflammatory factors IL-1β and IL-6, upregulation of anti-inflammatory IL-10 and VEGF growth factors, stimulation of collagen deposition, and promotion of granulation tissue and hair follicle regeneration. This research introduced a revolutionary concept for developing a nanozyme-based NIR photothermal system, which had great potential for bacterial infection-induced wound healing and skin tissue regeneration.

### Supplementary Information

**Additional file 1: Figure S1.** **a** TEM, **b** High resolution TEM, and **c** Selected area electron diffraction (SAED) images of CeO_2_ NPs. **Figure S2.** HAADF-STEM image of CeO_2_ NPs with related elemental mappings of O and Ce. **Figure S3.** a XPS spectrum of CeO_2_ NPs. (b) XPS spectrum of Ce 3d. **Figure S4.** XRD patterns for CeO_2_ NPs. **Figure S5.** Absorption spectrum of CeO_2_, F^-^, TMB, CeO_2_+F^-^, CeO_2_+TMB and CeO_2_+F^-^+TMB at pH 4 after 30 min of reaction. Concentration: CeO_2_ (30 µg/mL), F^-^ (5 mM), TMB (1 mM). The insert is the corresponding optical photograph (from left to right: CeO_2_, F^-^, TMB, CeO_2_+F^-^, CeO_2_+TMB and CeO_2_+F^-^+TMB). **Figure S6.** Optimizations of experiment condition. **a** Temperature change profiles of photothermal reaction with different concentrations of CeO_2_ NPs with or without fluoride ions. Effects of **b** pH and (C) NIR lamp power density. **Figure S7.** The absorption changes of CeO_2_+F^-^+TMB (CeO_2_: 30 µg/mL, F^-^: 5 mM, TMB: 1 mM) after the addition of GSH (1 mM) and 8-HQ (5 mM). **Figure S8.** Verification of the role of each module in the CeO_2_+F^-^+TMB system (CeO_2_: 30 µg/mL, F^-^: 5 mM, TMB: 1 mM). The concentrations of GSH and 8-HQ were 1 mM and 5 mM, respectively. **Figure S9.** Inhibition effect of CeO_2_+F^-^+TMB system. **Figure S10.** Selectivity investigation. All other anions concentrations were equal to the F^-^ concentration (5 mM). **Figure S11.** **a** Photographs of bacterial colonies formed by *E. coli* after exposure to acetate buffer (Control), F^-^, TMB, CeO_2_, CeO_2_+F^-^, CeO_2_+TMB and CeO_2_+F^-^+TMB without/with NIR irradiation. Concentration: CeO_2_ (30 µg/mL), F^-^ (400 µM), TMB (1 mM), acetate buffer (pH 4, 20 mM). **b** Statistical examination of survival rates of *E. coli* exposed to different samples without and with NIR irradiation. All data are presented as mean ± SD (n =3). **Figure S12.** **a** Colony photos of* E. coli *incubated with CeO_2_+TMB (CeO_2_: 30 µg/mL, TMB: 1 mM) with varying concentrations of fluoride ions (0, 10 µM, 50 µM, 200 µM, 400 µM) in the absence and presence of NIR light. (b) Statistical examination of survival rates of *E. coli* exposed to CeO_2_+TMB with varying fluoride ions concentrations without and with NIR irradiation. All data are presented as mean ± SD (n =3). **Figure S13.** The OD600 value of the supernatant as a function with the time after being treated by various conditions with *E. coli*. Concentration: CeO_2_ (30 µg/mL), F^-^ (400 µM), TMB (1 mM). All data are presented as mean ± SD (n =3). **Figure S14.** SEM images of *E. coli* samples after treatments with Control, CeO_2_+TMB, CeO_2_+F^-^+TMB without/with NIR irradiation (Scale bar: 500 nm). Concentration: CeO_2_ (30 µg/mL), F^-^ (400 µM), TMB (1 mM). **Figure S15.** Fluorescence images of *E. coli* samples after treatments with Control, CeO_2_+TMB, CeO_2_+F^-^+TMB without/with NIR irradiation (Scale bar: 20 μm). Concentration: CeO_2_ (30 µg/mL), F^-^ (400 µM), TMB (1 mM). **Figure S16.** Samples test for cytotoxicity after 24 h of co-culture with NIH/3T3 cells. All data are presented as mean ± SD (n =3). **Figure S17.** Hemolysis test on water, normal saline, CeO_2_, CeO_2_+F^-^, CeO_2_+TMB, CeO_2_+F^-^+TMB. The insert is the optical photograph. Concentration: CeO_2_ (30 µg/mL), F^-^ (400 µM), TMB (1 mM). **Figure S18.** **a** Infrared thermal pictures, and **b** the rise in temperature at the wound sites of mice at the designated periods under 808 nm NIR illumination (2.3 W/cm^2^) with treatments of CeO_2_+TMB (CeO_2_: 30 µg/mL, TMB: 1 mM). Error bars represent the standard deviation from the mean (n = 3). **Figure S19.** **a** Pictures depicting the progression of wound contraction from day 0 to day 12 in response to the treatments of CeO_2_+TMB + NIR group (808 nm, 2.3 W/cm^2^, 7 min). **b** The chart of the dynamic process of wound healing in CeO_2_+TMB + NIR group within 12 days. **c** Quantitative analysis of the wound size in CeO_2_+TMB + NIR group. Error bars represent the standard deviation from the mean (n = 3). **Figure S20.** The bacteria were separated from the wound tissue at different intervals and cultured on agar plates. **Figure S21.** **a** Pictures depicting the progression of wound contraction from day 0 to day 12 in response to penicillin G. **b** The chart of the dynamic process of wound healing within 12 days. **c** Quantitative analysis of the wound size within 12 days. **d** Comparison of wound size on day 12 with treatments of the penicillin G and CeO_2_+F^-^+TMB group. Error bars represent the standard deviation from the mean (n = 3). **Figure S22.** **a** H&E staining images of scar tissue thickness (Scale bar: the top of 200 µm and the bottom of 50 µm). **b** Quantification of scar tissue thickness. Error bars represent the standard deviation from the mean (n = 3). **Figure S23.** Body weights of different mice groups following treatment. All data are presented as mean ± SD (n =3). Concentration: CeO_2_ (30 µg/mL), F^-^ (400 µM), TMB (1 mM).

## Data Availability

All data analyzed during this study are included in this published article and its supplementary information fles.

## References

[1] Wei H, Wang E (2013). Nanomaterials with enzyme-like characteristics (nanozymes): next-generation artificial enzymes†. Chem Soc Rev.

[2] Yang W, Yang X, Zhu L, Chu H, Li X, Xu W (2021). Nanozymes: activity origin, catalytic mechanism, and biological application. Coord Chem Rev.

[3] Gao L, Zhuang J, Nie L, Zhang J, Zhang Y, Gu N, Wang T, Feng J, Yang D, Perrett S, Yan X (2007). Intrinsic peroxidase-like activity of ferromagnetic nanoparticles. Nat Nanotechnol.

[4] Jiang D, Ni D, Rosenkrans ZT, Huang P, Yan X, Cai W (2019). Nanozyme: new horizons for responsive biomedical applications. Chem Soc Rev.

[5] Wang H, Wan K, Shi X (2019). Recent advances in nanozyme research. Adv Mater.

[6] Wang Z, Zhang R, Yan X, Fan K (2020). Structure and activity of nanozymes: Inspirations for de novo design of nanozymes. Mater Today.

[7] Lei J, Yan H, Wu Y, Gu W, Zhu C, Du D, Lin Y (2020). When nanozymes meet single-atom catalysis. Angew Chem Int Ed.

[8] Lopez-Cantu DO, González-González RB, Melchor-Martínez EM, Martínez SAH, Araújo RG, Parra-Arroyo L, Sosa-Hernández JE, Parra-Saldívar R, Iqbal HMN (2022). Enzyme-mimicking capacities of carbon-dots nanozymes: properties, catalytic mechanism, and applications—a review. Int J Biol Macromol.

[9] Liu Q, Zhu X, Zhong L, Zhang S, Luo X, Liu Q, Tang L, Lu Y (2022). Recent advances in the applications of nanozymes for the efficient detection/removal of organic pollutants: a review. Environ. Sci Nano.

[10] Liu Q, Zhang A, Wang R, Zhang Q, Cui D (2021). A review on metal- and metal oxide-based nanozymes: properties mechanisms and applications. Nano-Micro Lett.

[11] Fisher RA, Gollan BG, Helaine S (2017). Persistent bacterial infections and persister cells. Nat Rev Microbiol.

[12] Westblade LF, Simon MS, Satlin MJ (2021). Bacterial coinfections in coronavirus disease 2019. Trends Microbiol.

[13] Cully M (2016). Antibacterial drugs: nosing around for new antibiotics. Nat Rev Drug Discov.

[14] York A (2017). Bacterial evolution: historical influences on antibiotic resistance. Nat Rev Microbiol.

[15] Zhao Y, Guo Q, Dai X, Wei X, Yu Y, Chen X, Li C, Cao Z, Zhang X (2019). A Biomimetic non-antibiotic approach to eradicate drug-resistant infections. Adv Mater.

[16] Xin Q, Shah H, Nawaz A, Xie W, Akram MZ, Batool A, Tian L, Jan SU, Boddula R, Guo B, Liu Q, Gong JR (2019). Antibacterial carbon-based nanomaterials. Adv Mater.

[17] Li B, Luo Y, Zheng Y, Liu X, Tan L, Wu S (2022). Two-dimensional antibacterial materials. Prog Mater Sci.

[18] Wang Y, Yang Y, Shi Y, Song H, Yu C (2020). Antibiotic-free antibacterial strategies enabled by nanomaterials: progress and perspectives. Adv Mater.

[19] Hao S, Han H, Yang Z, Chen M, Jiang Y, Lu G, Dong L, Wen H, Li H, Liu J, Wu L, Wang Z, Wang F (2022). Recent advancements on photothermal conversion and antibacterial applications over mxenes-based materials. Nano-Micro Lett.

[20] Sun Y, Liu J, Wang H, Li S, Pan X, Xu B, Yang H, Wu Q, Li W, Su X, Huang Z, Guo X, Liu H (2021). NIR laser-triggered microneedle-based liquid band-aid for wound care. Adv Funct Mater.

[21] Han Q, Lau JW, Do TC, Zhang Z, Xing B (2021). Near-infrared light brightens bacterial disinfection: recent progress and perspectives. ACS Appl Bio Mater.

[22] Hu H, Wang H, Yang Y, Xu J-F, Zhang X (2022). A bacteria-responsive porphyrin for adaptable photodynamic/photothermal therapy. Angew Chem Int Ed.

[23] Zhu X, Feng W, Chang J, Tan Y-W, Li J, Chen M, Sun Y, Li F (2016). Temperature-feedback upconversion nanocomposite for accurate photothermal therapy at facile temperature. Nat Commun.

[24] Cao C, Zhang T, Yang N, Niu X, Zhou Z, Wang J, Yang D, Chen P, Zhong L, Dong X, Zhao Y (2022). POD Nanozyme optimized by charge separation engineering for light/pH activated bacteria catalytic/photodynamic therapy. Signal Transduct Tar.

[25] Liu Y, Xu B, Lu M, Li S, Guo J, Chen F, Xiong X, Yin Z, Liu H, Zhou D (2022). Ultrasmall Fe-doped carbon dots nanozymes for photoenhanced antibacterial therapy and wound healing. Bioact Mater.

[26] Yin W, Yu J, Lv F, Yan L, Zheng LR, Gu Z, Zhao Y (2016). Functionalized nano-MoS_2_ with peroxidase catalytic and near-infrared photothermal activities for safe and synergetic wound antibacterial applications. ACS Nano.

[27] Jung HS, Verwilst P, Sharma A, Shin J, Sessler JL, Kim JS (2018). Organic molecule-based photothermal agents: an expanding photothermal therapy universe. Chem Soc Rev.

[28] Huang L, Li Y, Du Y, Zhang Y, Wang X, Ding Y, Yang X, Meng F, Tu J, Luo L, Sun C (2019). Mild photothermal therapy potentiates anti-PD-L1 treatment for immunologically cold tumors via an all-in-one and all-in-control strategy. Nat Commun.

[29] Hu B, Berkey C, Feliciano T, Chen X, Li Z, Chen C, Amini S, Nai MH, Lei Q-L, Ni R, Wang J, Leow WR, Pan S, Li Y-Q, Cai P, Miserez A, Li S, Lim CT, Wu Y-L, Odom TW, Dauskardt RH, Chen X (2020). Thermal-disrupting interface mitigates intercellular cohesion loss for accurate topical antibacterial therapy. Adv Mater.

[30] Yang Y, He P, Wang Y, Bai H, Wang S, Xu J-F, Zhang X (2017). Supramolecular radical anions triggered by bacteria in situ for selective photothermal therapy. Angew Chem Int Ed.

[31] Montini T, Melchionna M, Monai M, Fornasiero P (2016). Fundamentals and catalytic applications of CeO2-based materials. Chem Rev.

[32] Li J, Wen J, Li B, Li W, Qiao W, Shen J, Jin W, Jiang X, Yeung KWK, Chu PK (2018). Valence state manipulation of cerium oxide nanoparticles on a titanium surface for modulating cell fate and bone formation. Adv Sci.

[33] Xiao G, Li H, Zhao Y, Wei H, Li J, Su H (2022). Nanoceria-based artificial nanozymes: review of materials and applications. ACS Appl Nano Mater.

[34] He C, Ke Z, Liu K, Peng J, Yang Q, Wang L, Feng G, Fang J (2023). Nanozyme-based dual-signal sensing system for colorimetric and photothermal detection of AChE activity in the blood of liver-injured mice. Anal Bioanal Chem.

[35] Liu B, Huang Z, Liu J (2016). Boosting the oxidase mimicking activity of nanoceria by fluoride capping: rivaling protein enzymes and ultrasensitive F^-^ detection†. Nanoscale.

[36] Wang Y, Yang J, Zhao Y, Liu J (2019). Intentional hydrolysis to overcome the hydrolysis problem: detection of Ce(IV) by producing oxidase-like nanozymes with F^-^†. Chem Commun.

[37] Meng S, Yao Z, Liu J, Wang E, Li C, Jiang B, Xu Z (2022). Carbon dots capped cerium oxide nanoparticles for highly efficient removal and sensitive detection of fluoride. J Hazard Mater.

[38] Fu G, Sanjay ST, Zhou W, Brekken RA, Kirken RA, Li X (2018). Exploration of nanoparticle-mediated photothermal effect of TMB-H_2_O_2_ colorimetric system and its application in a visual quantitative photothermal immunoassay. Anal Chem.

[39] He L, Chen F, Zhang D, Xie S, Xu S, Wang Z, Zhang L, Cui C, Liu Y, Tan W (2020). Transducing complex biomolecular interactions by temperature-output artificial dna signaling networks. J Am Chem Soc.

[40] Du Y, Ke Z, Zhang J, Feng G (2022). Dual-signal output paper sensor based on coordinative self-assembly biomimetic nanozyme for point-of-care detection of biomarker. Biosens Bioelectron.

[41] Zhao M, Lin X, Zhou X, Zhang Y, Wu H, Liu Y (2022). Single probe-based chemical-tongue sensor array for multiple bacterial identification and photothermal sterilization in real time. ACS Appl Mater Interfaces.

[42] He X, Hao Y, Chu B, Yang Y, Sun A, Shi K, Yang C, Zhou K, Qu Y, Li H, Qian Z (2021). Redox-­activatable photothermal therapy and enzyme-mediated tumor starvation for synergistic cancer therapy. Nano Today.

[43] Zhu W, Liu Y, Liu P, Cao J, Shen A, Chu PK (2023). Blood-glucose-depleting hydrogel dressing as an activatable photothermal/chemodynamic antibacterial agent for healing diabetic wounds. ACS Appl Mater Interfaces.

