# Supplementary material for: Fluoride-activated photothermal system for promoting bacteria-infected wound healing
Source: J Nanobiotechnology. 2023 Sep 15;21:331. doi: 10.1186/s12951-023-02091-y (PMC10504784; doi:10.1186/s12951-023-02091-y)
Supplement: Supplementary file 1 — Additional file 1: Figure S1. a TEM, b High resolution TEM, and c Selected area electron diffraction (SAED) images of CeO2 NPs. Figure S2. HAADF-STEM image of CeO2 NPs with related elemental mappings of O and Ce. Figure S3. a XPS spectrum of CeO2 NPs. (b) XPS spectrum of Ce 3d. Figure S4. XRD patterns for CeO2 NPs. Figure S5. Absorption spectrum of CeO2, F-, TMB, CeO2+F-, CeO2+TMB and CeO2+F-+TMB at pH 4 after 30 min of reaction. Concentration: CeO2 (30 µg/mL), F- (5 mM), TMB (1 mM). The insert is the corresponding optical photograph (from left to right: CeO2, F-, TMB, CeO2+F-, CeO2+TMB and CeO2+F-+TMB). Figure S6. Optimizations of experiment condition. a Temperature change profiles of photothermal reaction with different concentrations of CeO2 NPs with or without fluoride ions. Effects of b pH and (C) NIR lamp power density. Figure S7. The absorption changes of CeO2+F-+TMB (CeO2: 30 µg/mL, F-: 5 mM, TMB: 1 mM) after the addition of GSH (1 mM) and 8-HQ (5 mM). Figure S8. Verification of the role of each module in the CeO2+F-+TMB system (CeO2: 30 µg/mL, F-: 5 mM, TMB: 1 mM). The concentrations of GSH and 8-HQ were 1 mM and 5 mM, respectively. Figure S9. Inhibition effect of CeO2+F-+TMB system. Figure S10. Selectivity investigation. All other anions concentrations were equal to the F- concentration (5 mM). Figure S11. a Photographs of bacterial colonies formed by E. coli after exposure to acetate buffer (Control), F-, TMB, CeO2, CeO2+F-, CeO2+TMB and CeO2+F-+TMB without/with NIR irradiation. Concentration: CeO2 (30 µg/mL), F- (400 µM), TMB (1 mM), acetate buffer (pH 4, 20 mM). b Statistical examination of survival rates of E. coli exposed to different samples without and with NIR irradiation. All data are presented as mean ± SD (n =3). Figure S12. a Colony photos of E. coli incubated with CeO2+TMB (CeO2: 30 µg/mL, TMB: 1 mM) with varying concentrations of fluoride ions (0, 10 µM, 50 µM, 200 µM, 400 µM) in the absence and presence of NIR light. (b) Statistical examinat [file 12951_2023_2091_MOESM1_ESM.docx]

**Additional file information**

**Fluoride-activated Photothefmal System For Promoting Bacteria-infected Wound Healing**

Yuanchun Du^b^^,#^, Zekai Liu^a,#^, Qingxin Yang^a^, Deshuai Zhen^c^,Yu Liu^b,^*, and Guangfu Feng^a,^*

^a^College of Bioscience and Biotechnology, Hunan Agricultural University, Changsha 410128, P. R. China

^b^State Key Laboratory of Chemo/Biosensing and Chemometrics, College of Chemistry and Chemical Engineering, Hunan University, Changsha 410082, P. R. China

^c^College of Public Health, Hengyang Medical School, University of South China, Hengyang 421001, P. R. China

^#^These authors contributed equally to this work

*Corresponding Authors: Yu Liu and Guangfu Feng

Email: yuliu0001@hnu.edu.cn (Y. Liu), [GuangfuFeng@hunau.edu.cn](mailto:GuangfuFeng@hunau.edu.cn) (G. Feng).

**Table of Content
1. Experimental section.……………………………………………….……..S3-S8**

**2. Supplemental Figures ..……………………………………………………S9-S22
3.** **References..……………………………..………………………………….S23**

**Experimental section**

**Reagents and instruments**

Sodium fluoride, sodium acetate, aluminum chloride, Glutathione reduced (GSH) and propidium iodide (PI) were acquired from Shanghai Macklin Biochemical Co., Ltd, China. Acetic acid, sodium chloride, sodium bromide, sodium iodide, sodium nitrate, sodium bicarbonate, sodium dihydrogen phosphate and 8-Hydroxyquinoline (8-HQ) were acquired from Sinopharm Chemical Reagent Co., Ltd, China. All additional analytical-grade reagents and solvents were obtained from commercial sources and used without further purification. All aqueous solutions were prepared with ultrapure water (18.2 MΩ·cm). Agar, Hoechst, 3, 3', 5, 5'-Tetramethylbenzidine (TMB), LB Broth and phosphate buffered solution (PBS) were obtained from Beijing Solarbio Science & Technology Co., Ltd, China. China's Multi Sciences (Lianke) Biotech Co., Ltd. supplied the Mouse IL-1β ELISA Kit, Mouse IL-6 ELISA Kit, Mouse IL-10 ELISA Kit, and Mouse VEGF ELISA Kit. Mice aged 10 to 13 weeks were acquired from Hunan Slike Jingda Laboratory Animal Co., Ltd.

The 808 nm laser was acquired from Changchun New Industries Optoelectronics Technology Co. The thermal imaging camera FLIR 3C was acquired from FLIR Systems. The pH readings were taken using a Bante 210 pH meter. At a voltage of 5.0 kV, scanning electron microscope (SEM) pictures were captured using a SU8100 field-emission electron microscope (Hitachi, Japan). Images captured with a transmission electron microscope (TEM) were obtained using JEOL 1011 equipment (JEOL, Japan). Field-emission transmission electron microscopy was used to describe elemental mapping results. Patterns of X-ray diffraction (XRD) was acquired using a Smart Lab X-ray powder diffractometer (Rigaku Co., Japan). X-ray photoelectron spectroscopy (XPS) measurement was acquired using a Thermo Scientific Escalab 250xi (Thermo Fisher, USA). A fluorescence microscope (Guangzhou Mingmei Photoelectric Technology, China) was applied to collect fluorescence images of bacteria. A UV-vis spectrophotometer (Shimadzu UV-3600Plus, Japan) was used to measure the UV-vis absorption.

**Synthesis of cerium dioxide and absorption measurements of TMB oxidation**

Cerium dioxide was synthesized based on previous literature reports [1]. Typically, CeO_2_ NPs (30 µg/mL) were disseminated in an acetate buffer (pH 4, 20 mM) containing predetermined amounts of fluoride ions, and then TMB (1 mM) was added to the reaction solution. After 30 minutes of incubation, the absorbances were measured using a UV-vis spectrophotometer.

**Photothermal performance testing**

1. Photothermal heating curve

Typically, CeO_2_ NPs (30 µg/mL) were disseminated in acetate buffer (pH 4, 20 mM) with varying concentrations of fluoride ions, followed by the addition of TMB (1 mM). The reaction solution was subjected to an 808 nm laser for about 100 seconds after 30 minutes of incubation, and the temperature change was recorded using a FLIR 3C thermal camera (FLIR 3C).

1. Photothermal cycle curve

Similar to the preceding process, the temperatures of the CeO_2_+F^-^+TMB system before and after laser irradiation were measured with the 808 NIR laser on for 100 seconds, followed by 10 minutes off for a photothermal cycle. This procedure was carried out five times.

1. Photothermal conversion efficiency

The following equations were used to compute the photothermal conversion efficiency [2]:

$$\theta=\frac{T_{\mathrm{surr}}-T_{(t)}}{T_{\mathrm{surr}}-T_{\max}}$$

$$t=-\frac{m_{d}C_{d}}{\mathrm{hA}}\ln\theta$$

$$\tau_{s}=\frac{m_{d}C_{d}}{\mathrm{hA}}$$

$$\eta=\frac{hA({\Delta T}_{max, mix}-{\Delta T}_{max,H_{2}O})}{I(1-{10}^{{-A}_{\lambda}})}$$

where η was the photothermal conversion efficiency; *h* was the heat transfer coefficient; *A* was the surface area of the container; ${\Delta T}_{max,mix}$ was the temperature change of the mixture at the maximum steady-state temperature; ${\Delta T}_{max,H_{2}O}$ was the temperature change of water at the maximum steady-state temperature; I was the laser power (2.3 W/cm^2^); $A_{\lambda}$ was the absorbance of the mixture after 30 min of reaction at 808 nm; $T_{surr}$ was the ambient temperature of the surroundings; $T_{(t)}$ was the solution temperature; $T_{max}$ was the equilibrium temperature of the mixture; $m_{d}$ was the mass of H_2_O (0.1×10^-3^ kg); and $C_{d}$ was the heat capacity of H_2_O (4.2×10^3^ J/kg/s). The time constant ($\tau_{s}$) was calculated from the slope of the linear plot of time (t) *versus* -ln(θ) in the sample cooling system. Then, the value of *hA* could be calculated. Every parameter was clear, and the photothermal conversion efficiency (η) could be calculated.

**Temperature readings against the various fluoride ions concentrations**

CeO_2_ NPs (30 g/mL) were disseminated in an EP tube containing acetate buffer (pH 4, 20 mM) with different concentrations of fluoride ions (0, 1 µM, 5 µM, 10 µM, 25 µM, 50 µM, 100 µM, 200 µM, 300 µM, 400 µM, 500 µM, 600 µM, 800 µM, 1000 µM, 2000 µM, 3000 µM, 4000 µM, 5000 µM), followed by the addition of TMB (1 mM) to the reaction solution. After 30 minutes of incubation, the EP tube was exposed to light (808 nm, 2.3 W/cm^2^) for 100 seconds, and the temperature was recorded using a thermal imaging camera.

**In vitro antibacterial assay**

The agar plate technique was used to evaluate the antibacterial capacity. Separately, *S. aureus* and *E. coli* were cultured with 14 distinct groups: (1) Control (acetate buffer), (2) Control + NIR, (3) F^-^, (4) F^-^ + NIR, (5) TMB, (6) TMB + NIR, (7) CeO_2_, (8) CeO_2_ + NIR, (9) CeO_2_+F^-^, (10) CeO_2_+ F^-^ + NIR, (11) CeO_2_+TMB, (12) CeO_2_+TMB + NIR, (13) CeO_2_+F^-^+TMB, (14) CeO_2_+F^-^+TMB + NIR. Concentration: CeO_2_ (30 µg/mL), F^-^ (400 µM), TMB (1 mM), acetate buffer (pH 4, 20 mM). After incubation for 30 min, groups 2, 4, 6, 8, 10, 12, and 14 were further exposed to 808 nm laser (2.3 W/cm^2^) for 7 min. The bacterial suspensions were spread on the LB Broth agar plates. The *S. aureus* colonies and *E. coli* colonies were counted after about 18 hours of incubation at 37 °C. All experiments were repeated three times. The following equation was used to compute the survival rate of bacteria [3]:

Survival rate (%) = CFU_exp_ / CFU_ctrl_

CFU_exp_ is the Colony-Forming Unit of the experimental group, and CFU_ctrl_ is the Colony-Forming Unit of the control group.

To further evaluate the relationship between the concentration of fluoride ions on the survival rate of *S.aureus* and *E.coli*, CeO_2_ NPs (30 µg/mL) was disseminated in acetate buffer (pH 4, 20 mM) containing a variety of fluoride ions (0, 10 µM, 50 µM, 200 µM, 400 µM) in EP tubes before TMB (1 mM) was added to the reaction mixture. The EP tubes were either subjected to or not exposed to NIR irradiation (808 nm, 2.3 W/cm^2^, 7 min). The bacterial suspensions were finally spread on LB Broth agar plates. The *S. aureus* and *E. coli* colonies were counted after about 18 hours of incubation at 37 °C. Each experiment was conducted three times.

**SEM characterization of bacteria**

The bacterial morphology was monitored using a scanning electron microscope (SEM) before and after exposure to NIR light. The treated bacterial (*S. aureus* and *E. coli*) were cultured with 6 distinct groups: (1) Control, (2) Control + NIR, (3) CeO_2_+TMB, (4) CeO_2_+TMB + NIR, (5) CeO_2_+ F^-^+TMB, (6) CeO_2_+F^-^+TMB + NIR. Centrifugation was used to separate the treated bacteria from the supernatant, which was then discarded (8,000 rpm, 5 min). Afterward, the sediment was washed with PBS. The bacteria were then subjected to a 4-hour fixation with 1 mL of a 2.5% glutaraldehyde solution, followed by gradient dehydration with a series of ethanol solutions (70%, 80%, 90%, and 100%, 15 minutes each step). The samples were finally coated with platinum by sputtering prior to SEM analysis after being dried.

**Live/dead fluorescent staining of bacteria**

Like the above procedure, groups of (1) Control (acetate buffer), (2) Control + NIR, (3) CeO_2_+TMB, (4) CeO_2_+TMB+NIR, (5) CeO_2_+F^-^+TMB, (6) CeO_2_+F^-^+TMB + NIR were treated with *S. aureus* and *E. coli*. The treated bacteria were separated by centrifugation (8000 rpm for 5 minutes) and the supernatant was disposed of. Bacteria were stained with fluorescent live/dead staining dyes, PI (0.1 mg) and Hoechst 33342 (0.1 mg), in 100 µL of normal saline. After 30 minutes in a dark bath of cold water, the bacteria were washed three times with PBS before being diluted in normal saline (100 µL). The living/dead microorganisms were analyzed using a fluorescence microscope.

**In vivo antibacterial activity and wound healing efficiency**

To evaluate the antibacterial effectiveness of the CeO_2_+F^-^+TMB system in vivo, male mice (11-13 weeks) with wound infections were utilized as a model. On the back of each mouse, an 8-millimeter-diameter wound was first created. To generate the local infection mouse model, a wound was injected with *S. aureus* suspension (1x10^8^ CFU/mL). After an hour, mice were divided into four distinct groups: the control group (acetate buffer) without and with NIR irradiation (808 nm, 2.3 W/cm^2^), CeO_2_+F+TMB group without and with NIR irradiation. The FLIR C3 thermal imaging camera captured temperature and infrared images every minute in real-time. Digital images of the wounds were collected at predetermined intervals (days 0, 4, 8 and 12).

To demonstrated the role of F^-^ in CeO_2_+F^-^+TMB + NIR photothermal system, male mice (11-13 weeks) with wound infections were utilized as a model. On the back of each mouse, an 8-millimeter-diameter wound was first created. To generate the local infection mouse model, a wound was injected with *S. aureus* suspension (1x10^8^ CFU/mL). After an hour, the CeO_2_ +TMB group was treated by NIR irradiation (808 nm, 2.3 W/cm^2^). The FLIR C3 thermal imaging camera captured temperature and infrared images every minute in real-time. Digital images of the wounds were collected at predetermined intervals (days 0, 4, 8 and 12).

On day 8, the skin tissue samples of the control group (acetate buffer) without and with NIR irradiation (808 nm, 2.3 W/cm^2^), CeO_2_+F^-^+TMB group without and with NIR irradiation were obtained for H&E, Masson's trichrome, immunohistochemical (IL-1β, IL-6, and IL-10) and growth factor (VEGF) analyses. In addition, wound exudate was collected, diluted, and disseminated on agar plates at two hours and seven days. The bacterial colony was photographed using a smartphone following 18 hours of 37 °C incubation. Moreover, the daily body weight of each rat was determined using an electronic balance.

**In vivo wound healing efficiency of antibiotic**

On the back of each mouse (11-13 weeks), an 8-millimeter-diameter wound was created. To generate the local infection mouse model, a wound was injected with S. aureus suspension (1x10^8^ CFU/mL). After an hour, 100 μL of fresh ampicillin (250 μg/mL) was injected into the wound. Digital images of the wounds were collected at predetermined intervals (days 0, 4, 8 and 12).

**In Vivo Biosafety**

1. **Cell cytotoxicity assay**

The CCK-8 test was used to assess the vitality of the NIH3T3 fibroblasts after one day of culture. After 24 hours of exposure to the CeO_2_, CeO_2_+F^-^, CeO_2_+TMB and CeO_2_+F^-^+TMB, CCK-8 solution (100 µL) was added to each well. After 4 hours of incubation, a UV-vis spectrophotometer reading was taken to measure the cell cytotoxicity.

1. **Hemolytic activity test**

Fresh mouse blood with 3% sodium citrate was spun for five minutes at 3,000 rpm to separate the red blood, which was then thoroughly removed from the supernatant using PBS. The acquired red blood cells (40 µL) were dissolved in related material (960 µL) in an EP tube to generate a homogenous dispersion. The combination of ultrapure water (960 µL) and red blood cell suspension (40 µL) served as the PC, whereas normal saline (960 µL) was employed as the NC. The EP tubes were then submerged in 37 °C water for 90 minutes and centrifuged (3,000 rpm, 5 min). The absorbance of the supernatant at 545 nm was used to determine the hemolytic activity. The ratio of hemolysis is computed using the following formula [3]:

hemolysis ratio (%) = (A_sample_ - A_NC_) / (A_PC_ - A_NC_) × 100 %

1. **In vivo toxicology studies**

The mice received therapy for 12 days before being euthanized to collect blood serum samples for biochemical examination. At this point, vital organs containing the heart, liver, spleen, lung, and kidney were extracted for H&E staining.


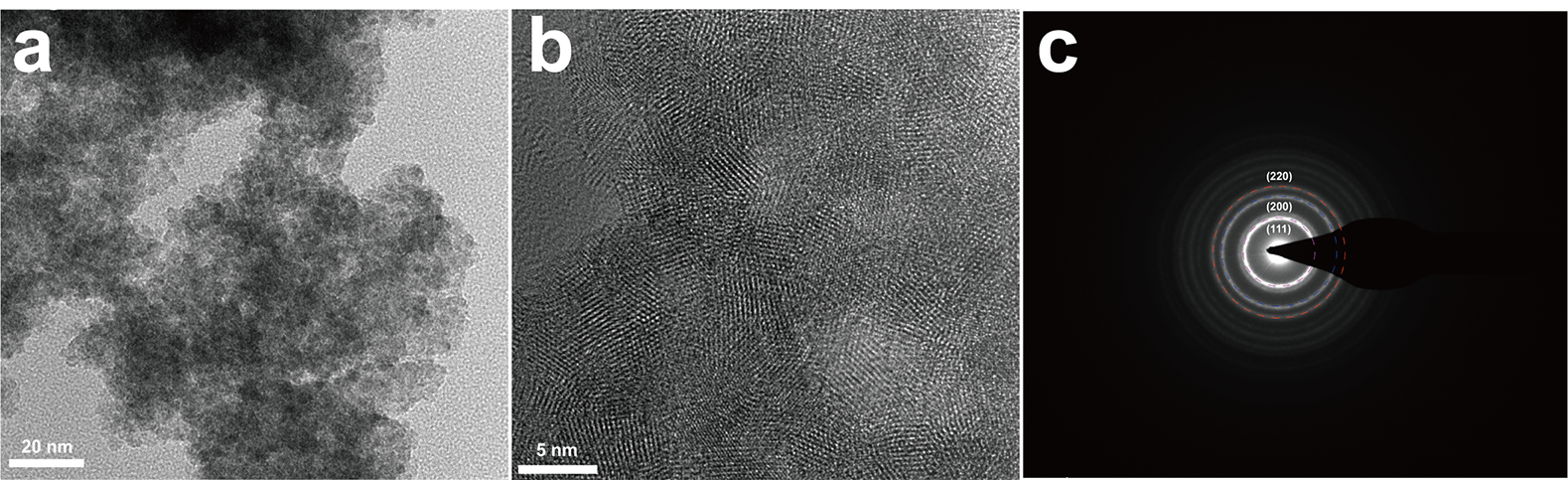
Figure S1 (a)TEM, (b) High resolution TEM, and (c) Selected area electron diffraction (SAED) images of CeO_2_ NPs.


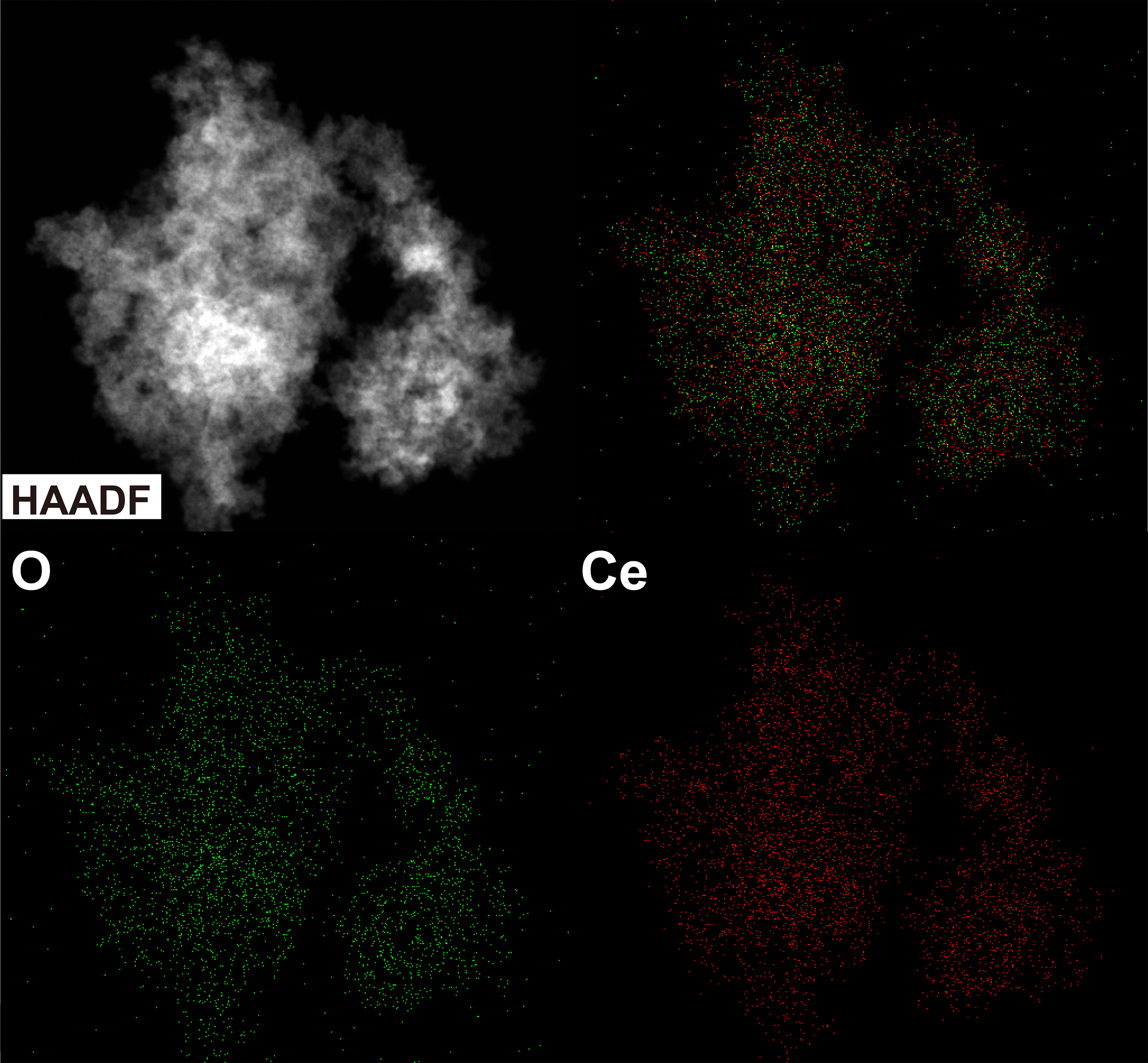


Figure S2 HAADF-STEM image of CeO_2_ NPs with related elemental mappings of O and Ce.


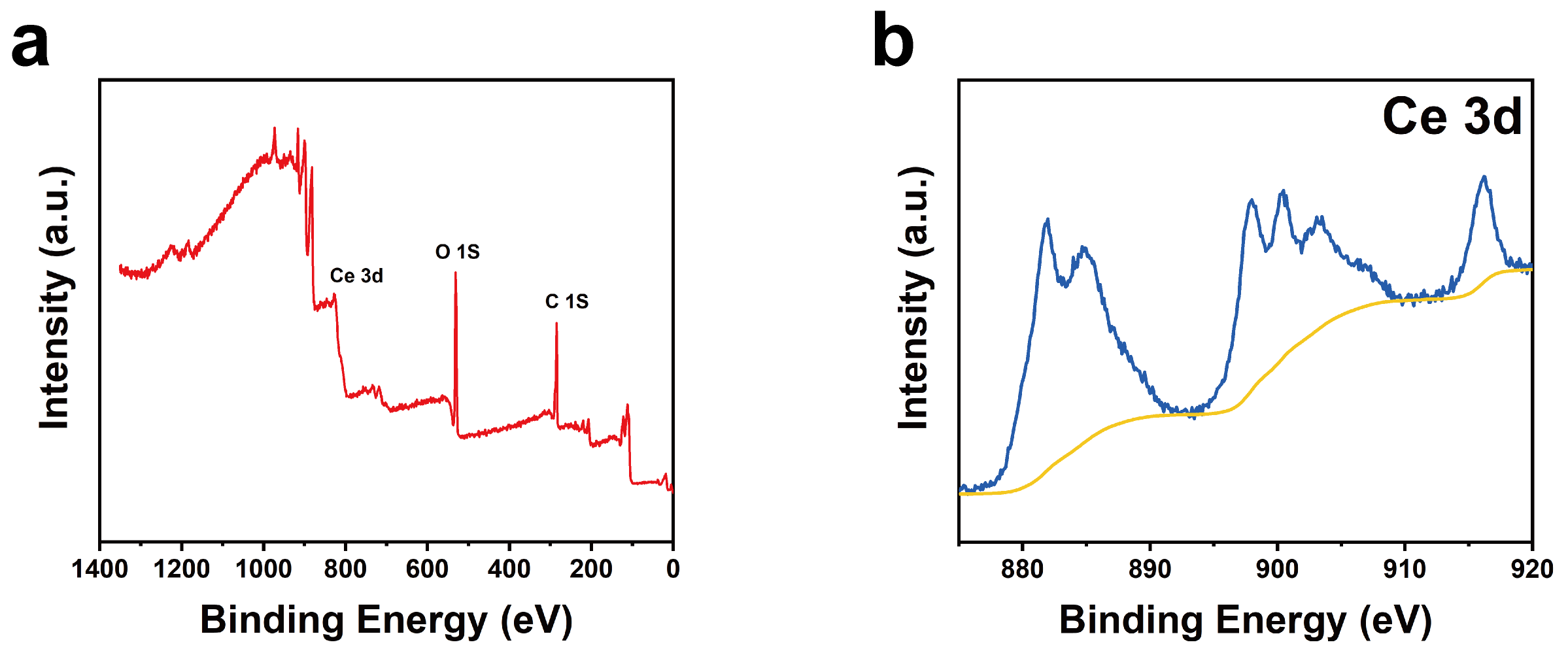


Figure S3 (a) XPS spectrum of CeO_2_ NPs. (b) XPS spectrum of Ce 3d.





Figure S4 XRD patterns for CeO_2_ NPs.


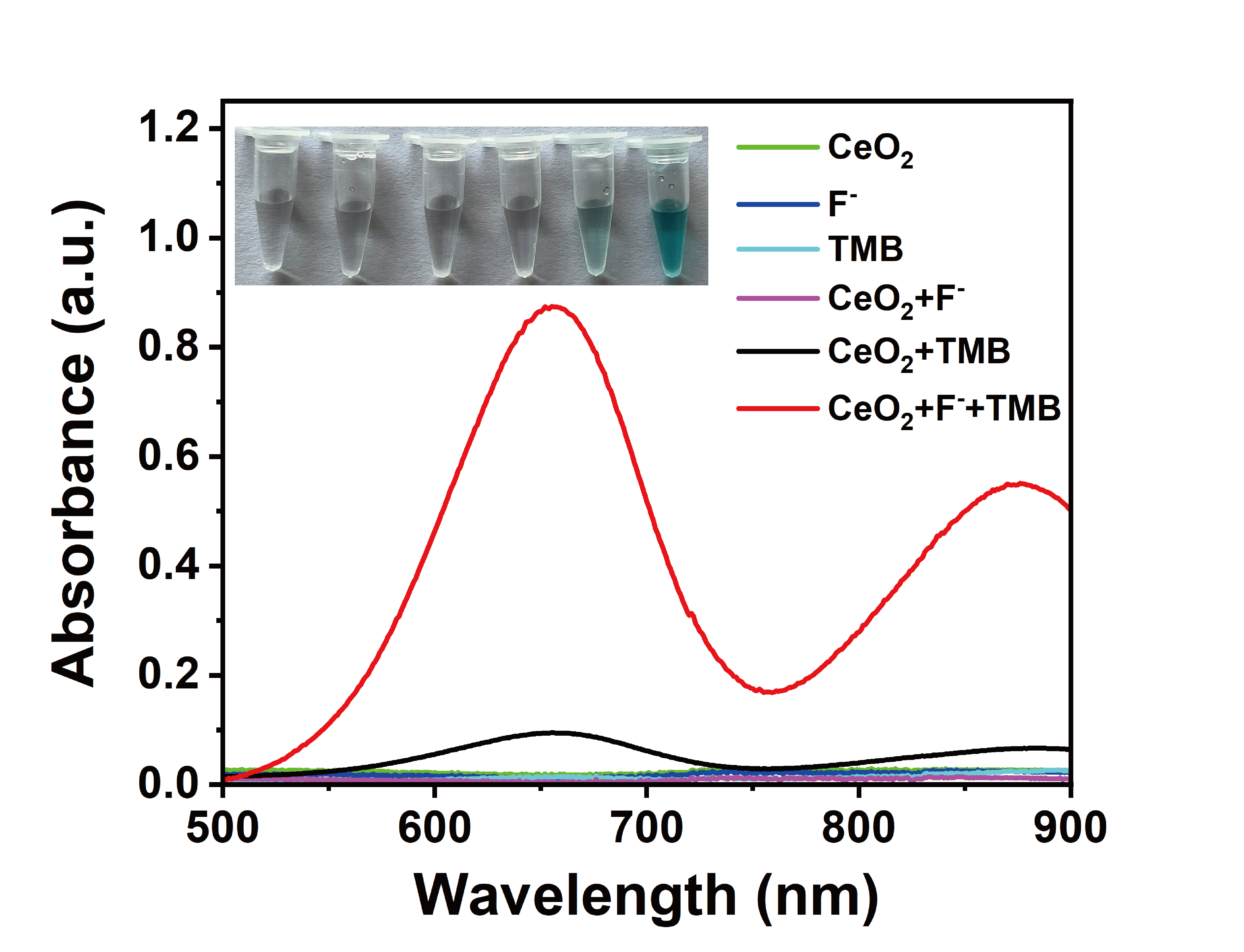


Figure S5 Absorption spectrum of CeO_2_, F^-^, TMB, CeO_2_+F^-^, CeO_2_+TMB and CeO_2_+F^-^+TMB at pH 4 after 30 min of reaction. Concentration: CeO_2_ (30 µg/mL), F^-^ (5 mM), TMB (1 mM). The insert is the corresponding optical photograph (from left to right: CeO_2_, F^-^, TMB, CeO_2_+F^-^, CeO_2_+TMB and CeO_2_+F^-^+TMB).


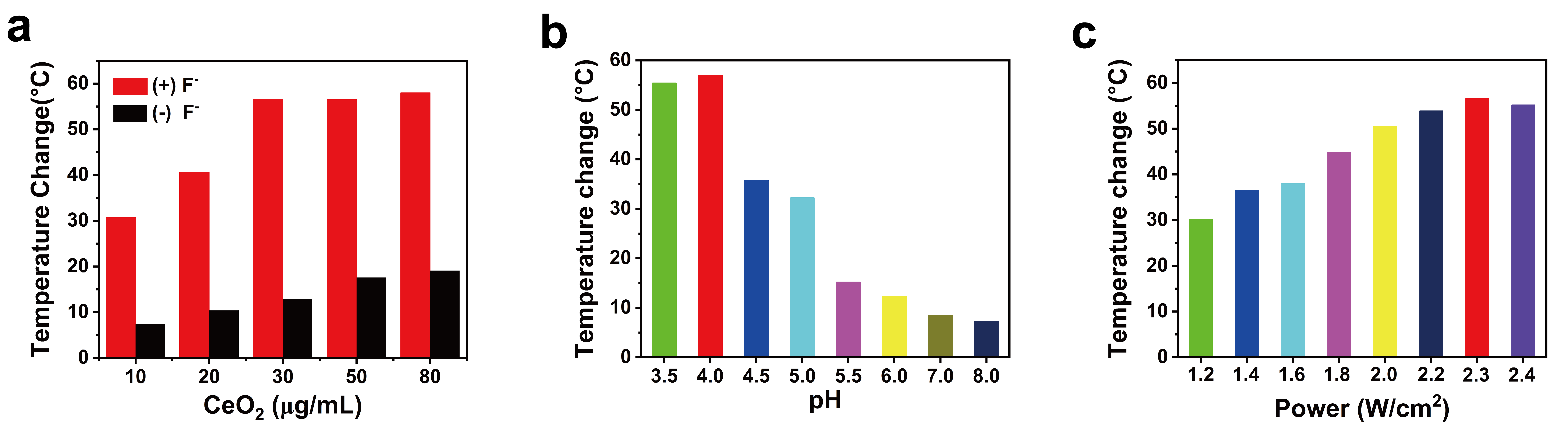


Figure S6 Optimizations of experiment condition. (a) Temperature change profiles of photothermal reaction with different concentrations of CeO_2_ NPs with or without fluoride ions. Effects of (b) pH and (C) NIR lamp power density.


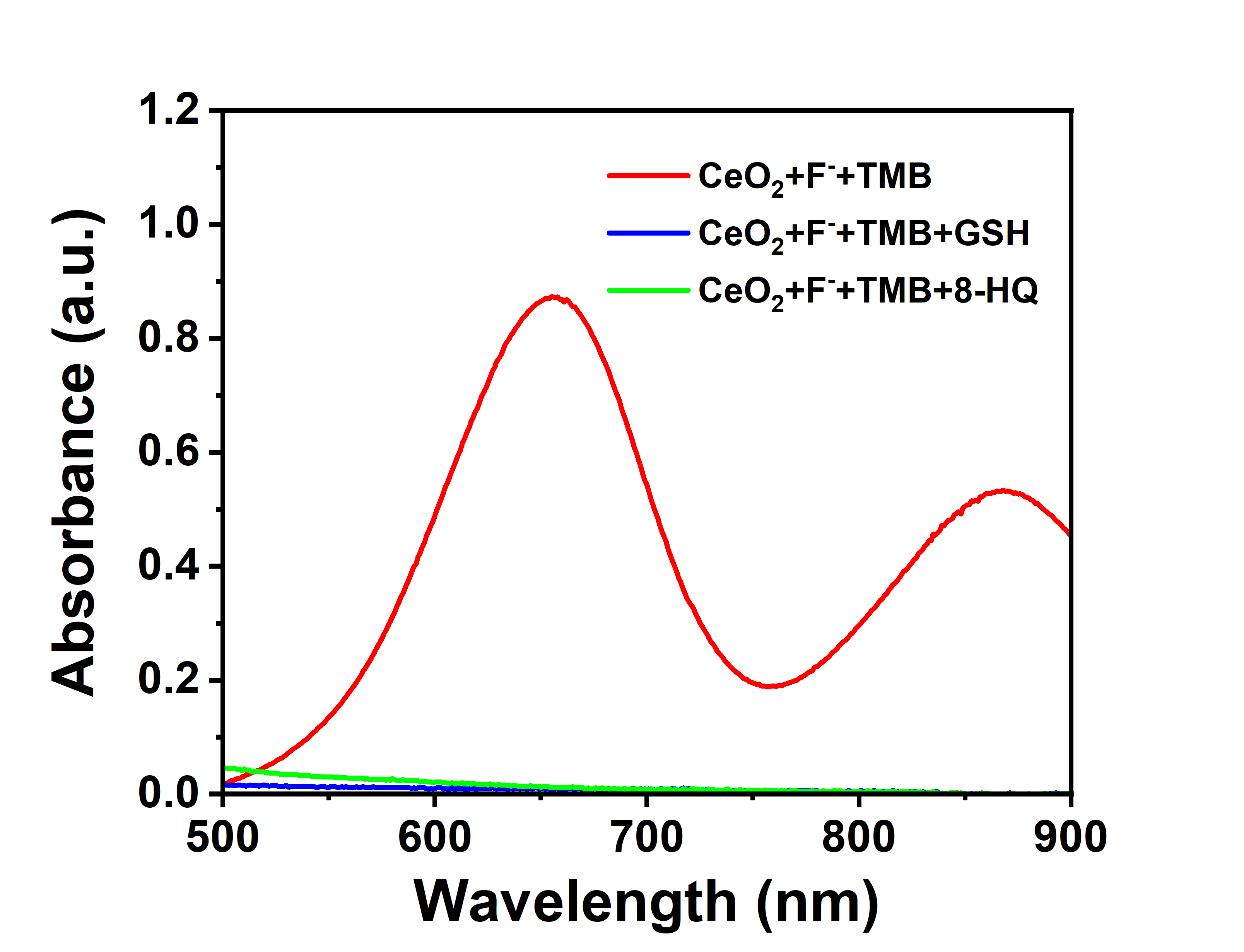


Figure S7 The absorption changes of CeO_2_+F^-^+TMB (CeO_2_: 30 µg/mL, F^-^: 5 mM, TMB: 1 mM) after the addition of GSH (1 mM) and 8-HQ (5 mM).


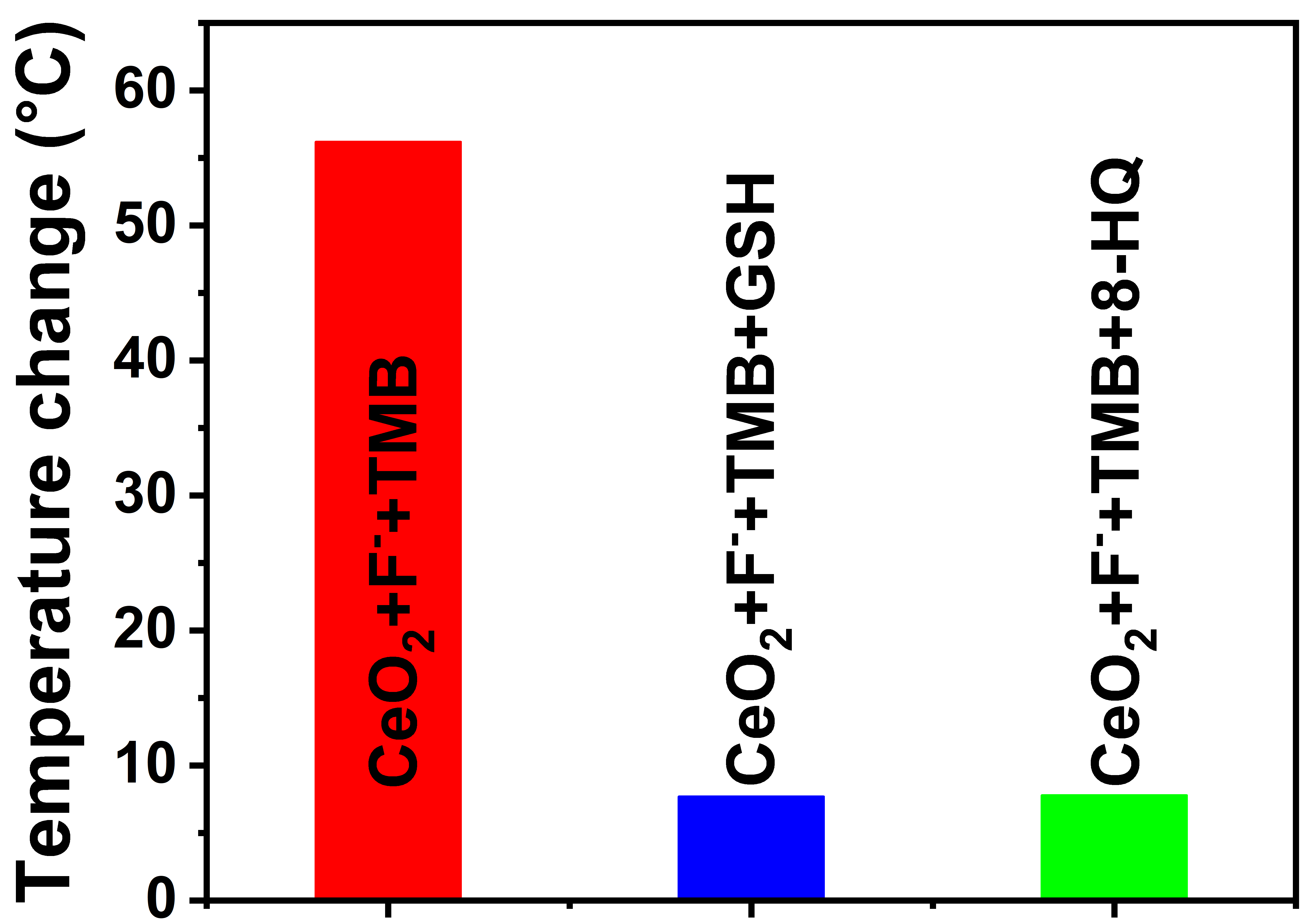


Figure S8 Verification of the role of each module in the CeO_2_+F^-^+TMB system (CeO_2_: 30 µg/mL, F^-^: 5 mM, TMB: 1 mM). The concentrations of GSH and 8-HQ were 1 mM and 5 mM, respectively.


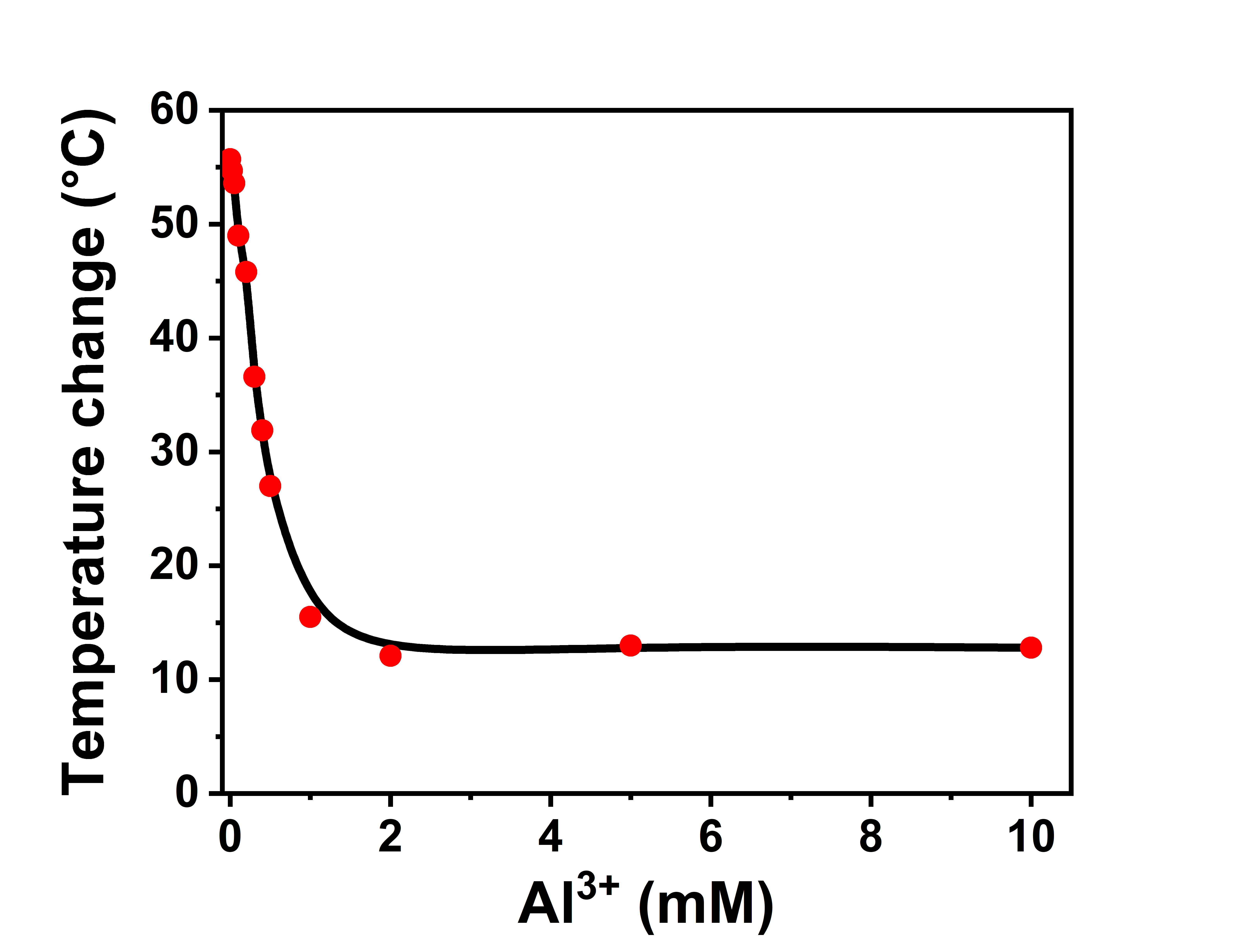


Figure S9 Inhibition effect of CeO_2_+F^-^+TMB system (CeO_2_: 30 µg/mL, F^-^: 5 mM, TMB: 1 mM) incubation with different concentrations of Al^3+^ (0 mM, 0.02 mM, 0.05 mM, 0.1 mM, 0.2 mM, 0.3 mM, 0.4 mM, 0.5 mM, 1 mM, 2 mM, 5 mM, 10 mM).


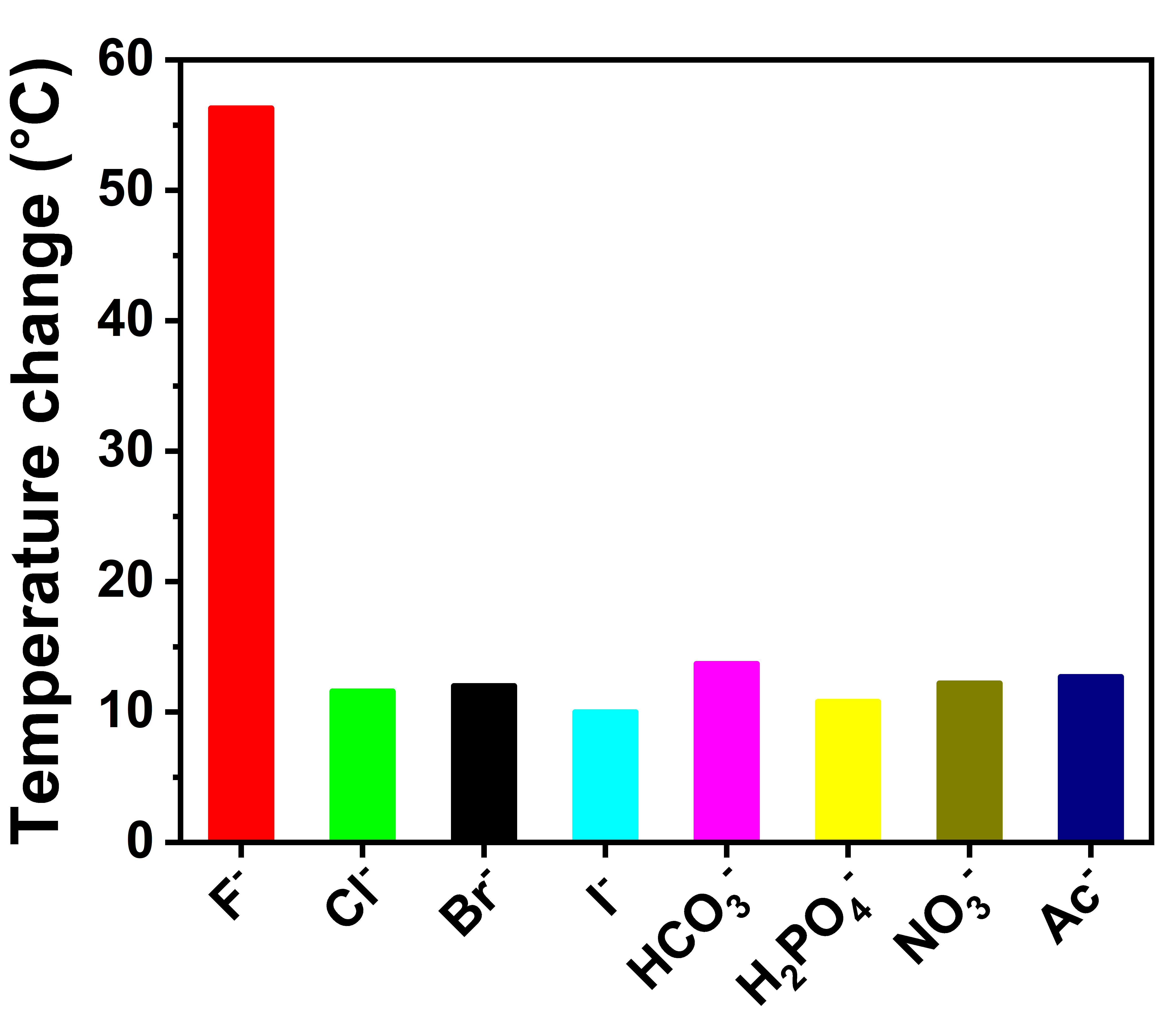


Figure S10 Selectivity investigation. All other anions concentrations were equal to the F^-^ concentration (5 mM).


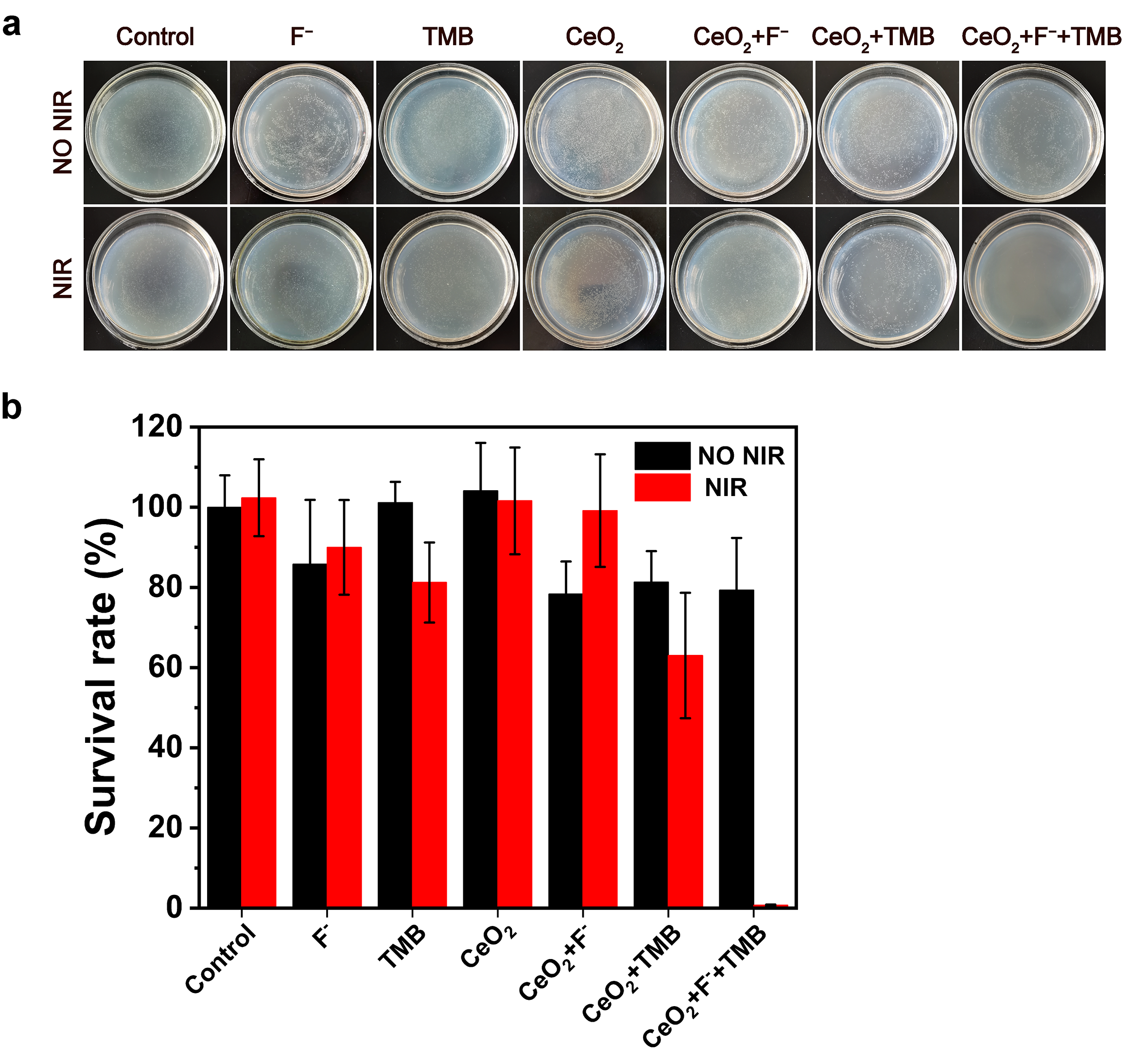


Figure S11 (a) Photographs of bacterial colonies formed by *E. coli* after exposure to acetate buffer (Control), F^-^, TMB, CeO_2_, CeO_2_+F^-^, CeO_2_+TMB and CeO_2_+F^-^+TMB without/with NIR irradiation. Concentration: CeO_2_ (30 µg/mL), F^-^ (400 µM), TMB (1 mM), acetate buffer (pH 4, 20 mM). (b) Statistical examination of survival rates of *E. coli* exposed to different samples without and with NIR irradiation. All data are presented as mean ± SD (n =3).


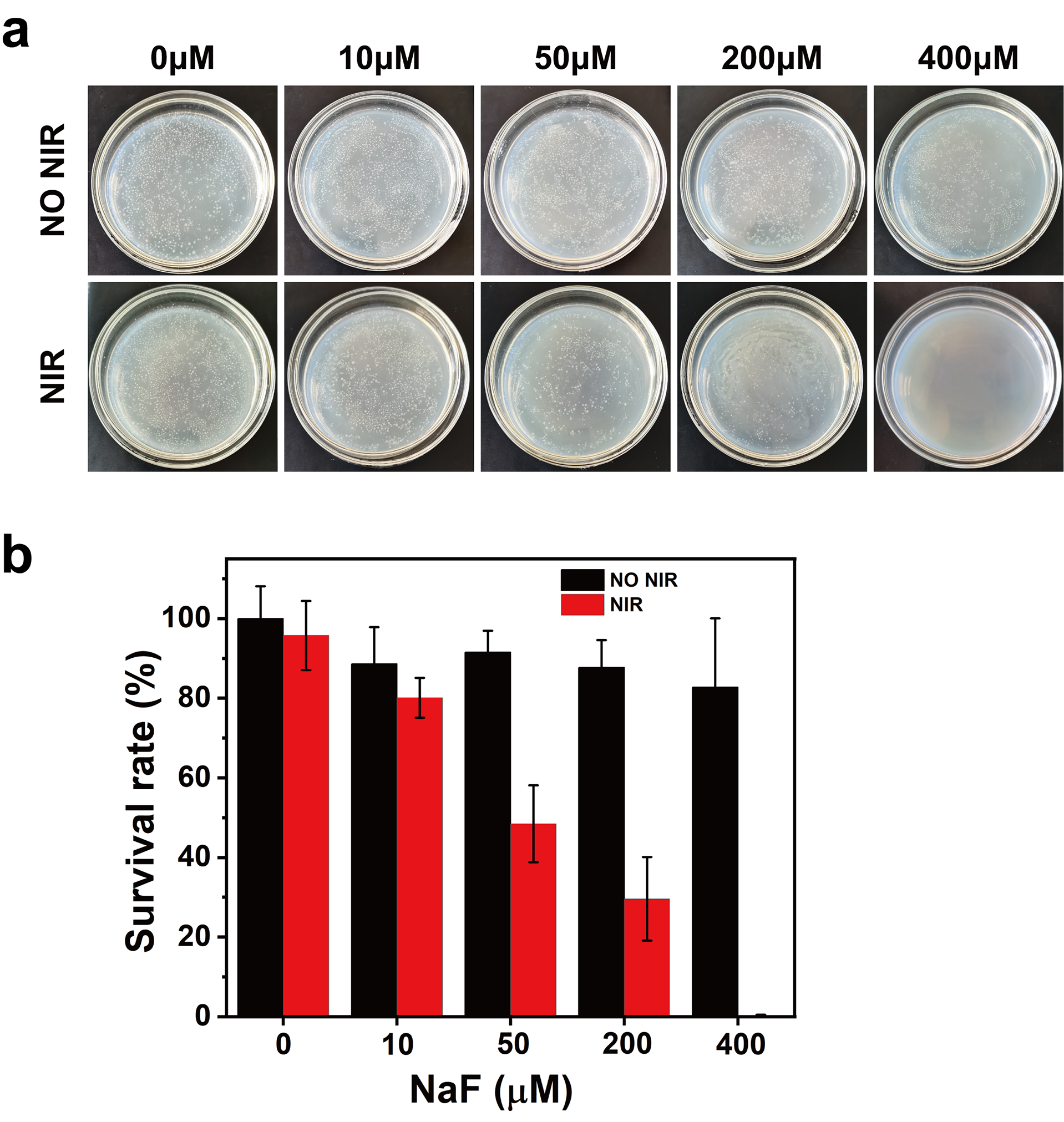


Figure S12 (a) Colony photos of *E. coli* incubated with CeO_2_+TMB (CeO_2_: 30 µg/mL, TMB: 1 mM) with varying concentrations of fluoride ions (0, 10 µM, 50 µM, 200 µM, 400 µM) in the absence and presence of NIR light. (b) Statistical examination of survival rates of *E. coli* exposed to CeO_2_+TMB with varying fluoride ions concentrations without and with NIR irradiation. All data are presented as mean ± SD (n =3).


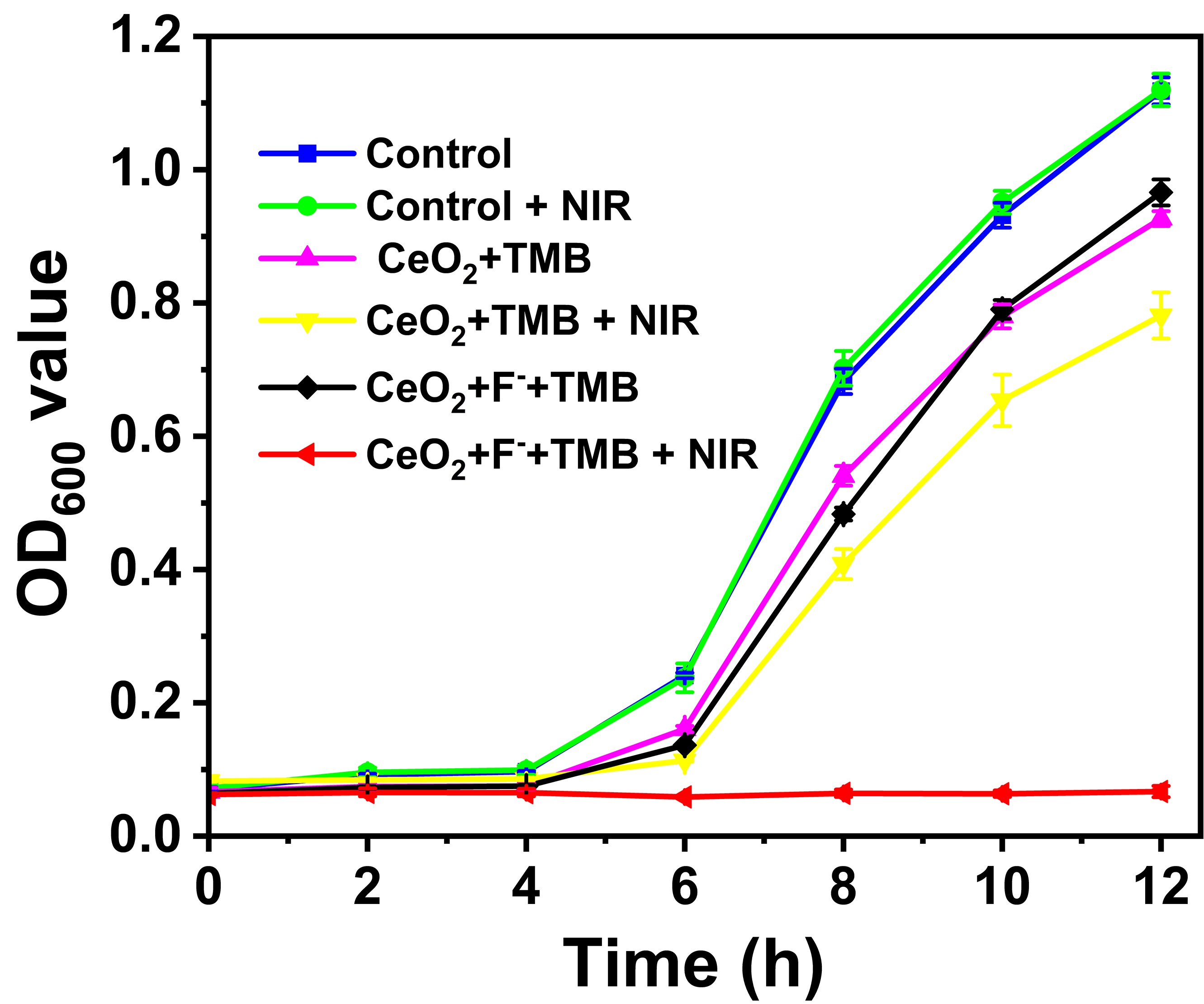


Figure S13 The OD600 value of the supernatant as a function with the time after being treated by various conditions with *E. coli*. Concentration: CeO_2_ (30 µg/mL), F^-^ (400 µM), TMB (1 mM). All data are presented as mean ± SD (n =3).


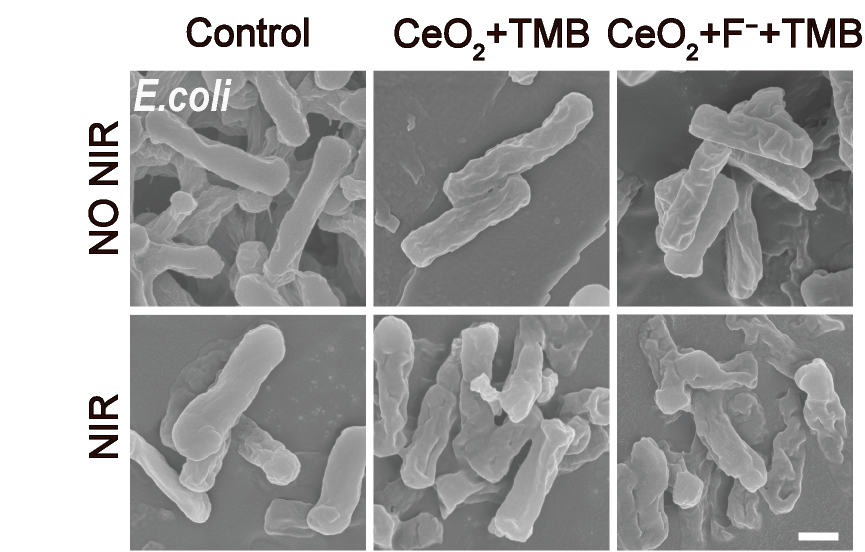


Figure S14 SEM images of *E. coli* samples after treatments with Control, CeO_2_+TMB, CeO_2_+F^-^+TMB without/with NIR irradiation (Scale bar: 500 nm). Concentration: CeO_2_ (30 µg/mL), F^-^ (400 µM), TMB (1 mM).


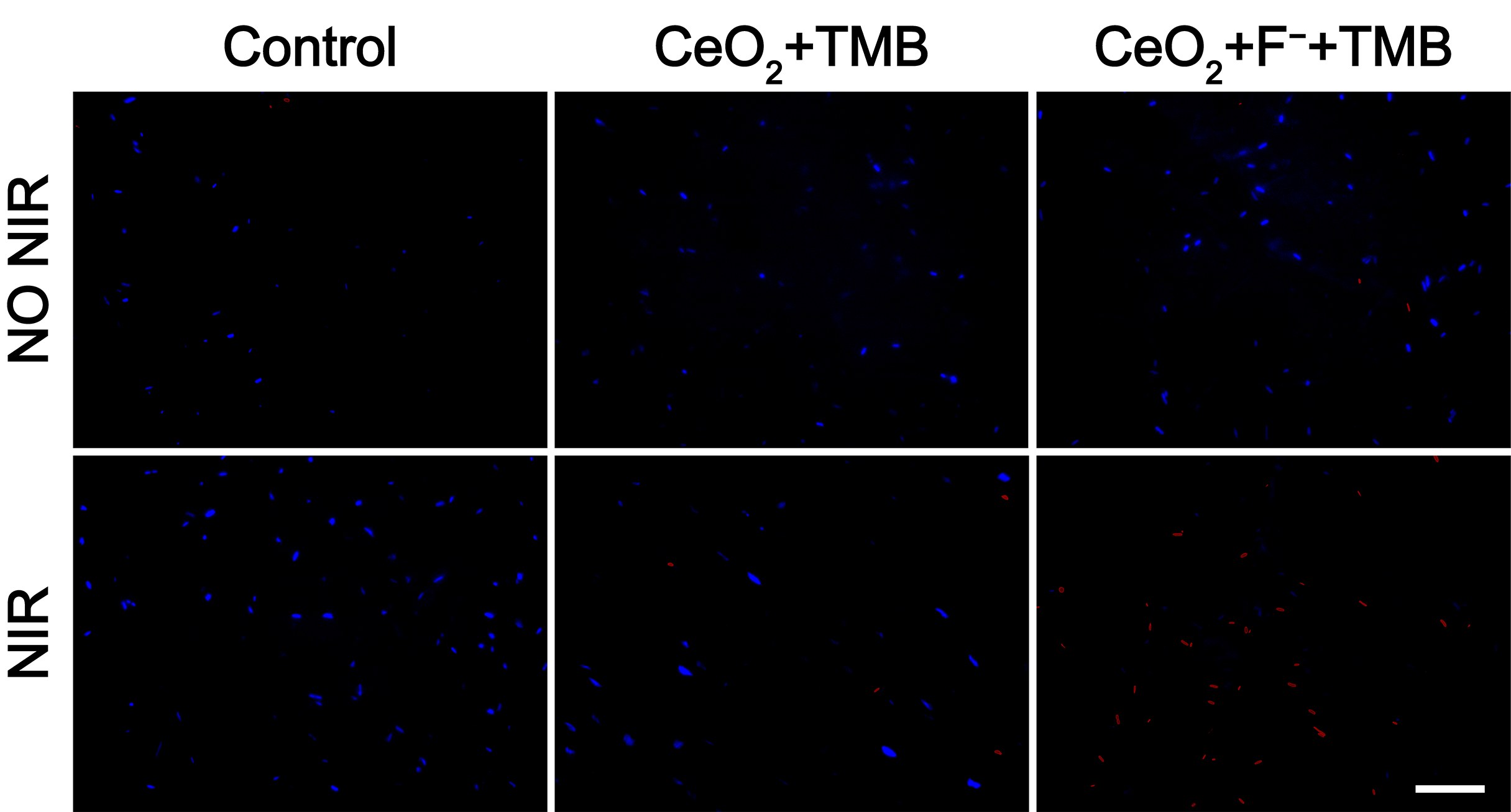


Figure S15 Fluorescence images of *E. coli* samples after treatments with Control, CeO_2_+TMB, CeO_2_+F^-^+TMB without/with NIR irradiation (Scale bar: 20 μm). Concentration: CeO_2_ (30 µg/mL), F^-^ (400 µM), TMB (1 mM).


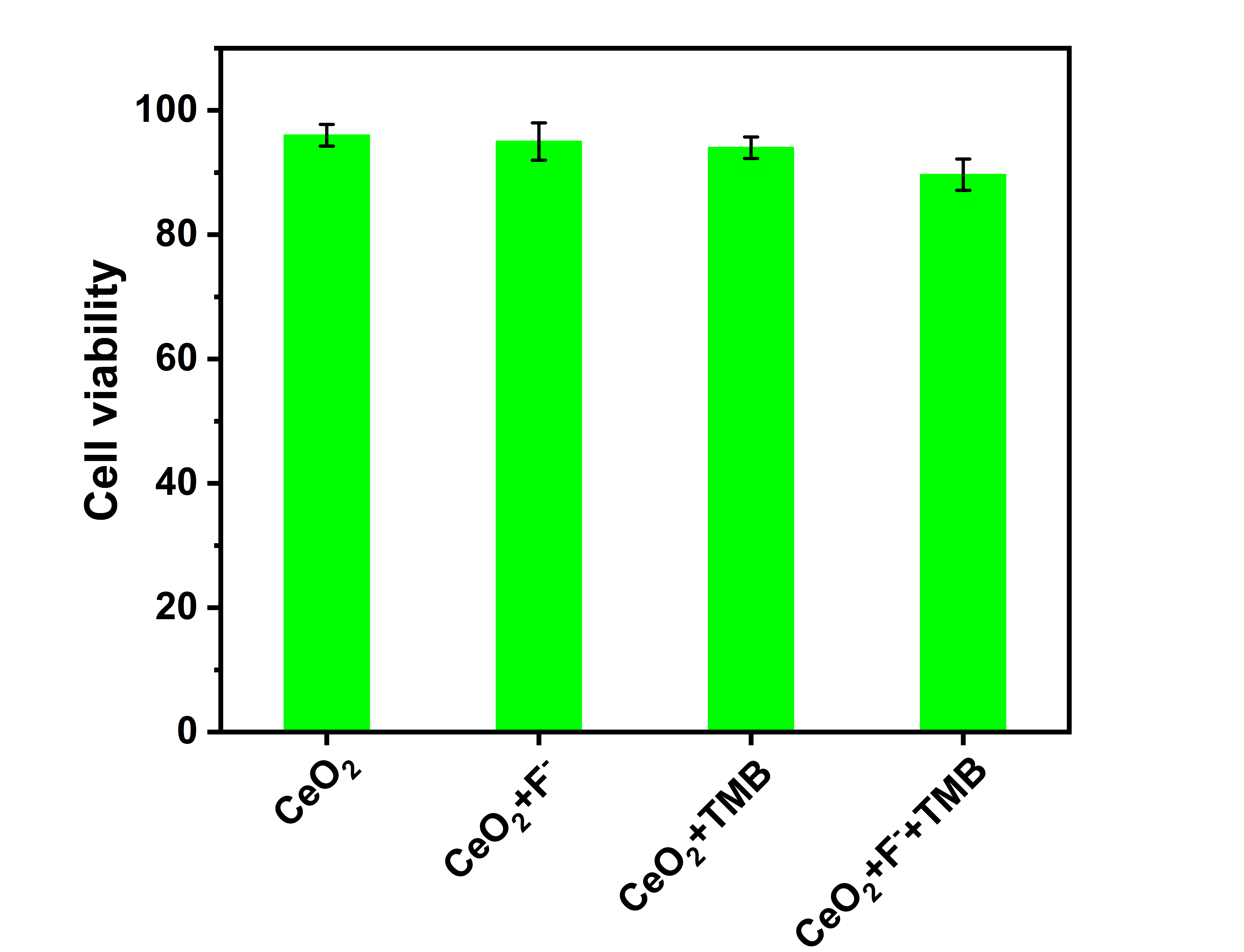


Figure S16 Samples test for cytotoxicity after 24h of co-culture with NIH/3T3 cells. All data are presented as mean ± SD (n =3).


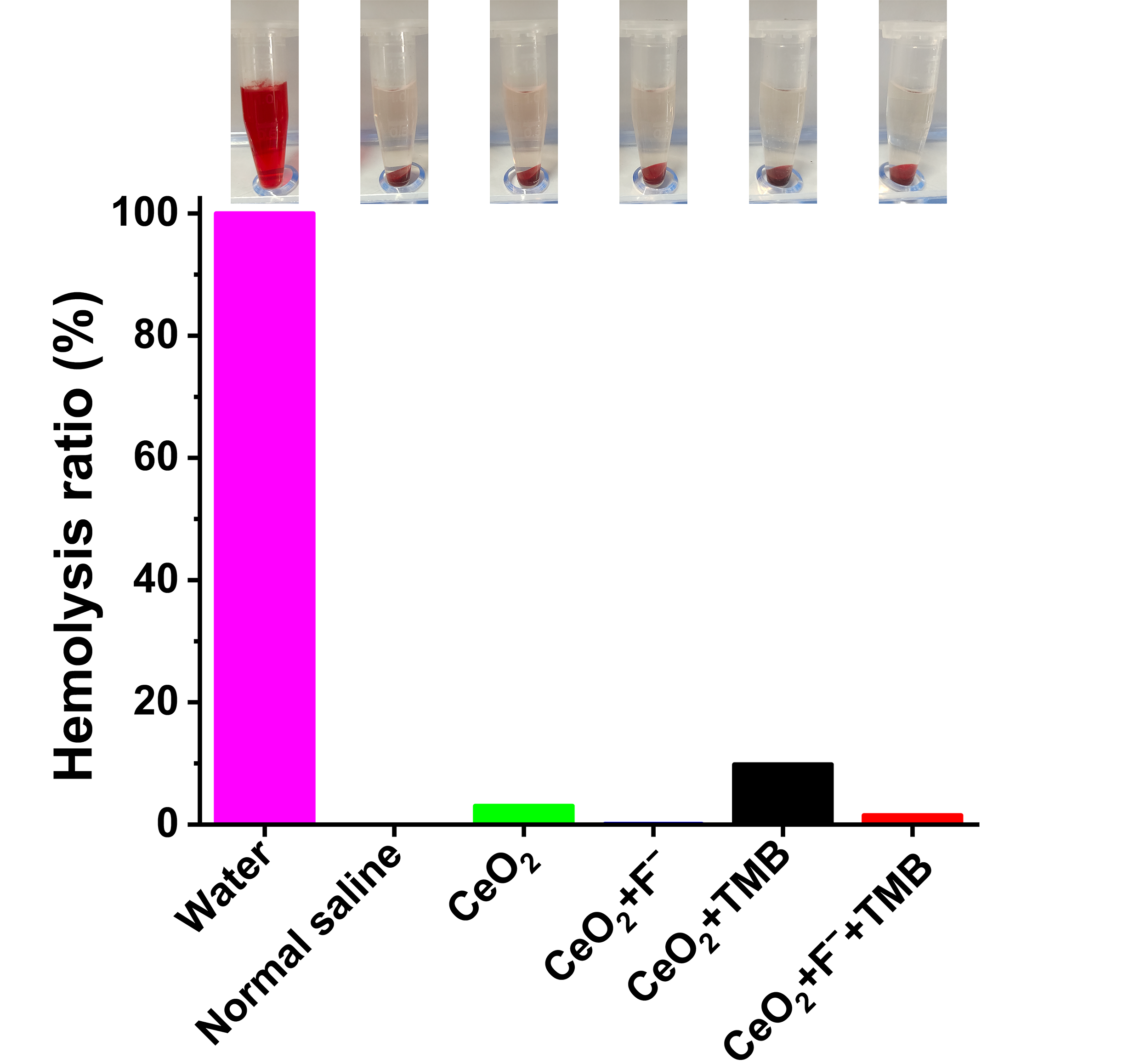


Figure S17 Hemolysis test on water, normal saline, CeO_2_, CeO_2_+F^-^, CeO_2_+TMB, CeO_2_+F^-^+TMB. The insert is the optical photograph. Concentration: CeO_2_ (30 µg/mL), F^-^ (400 µM), TMB (1 mM).


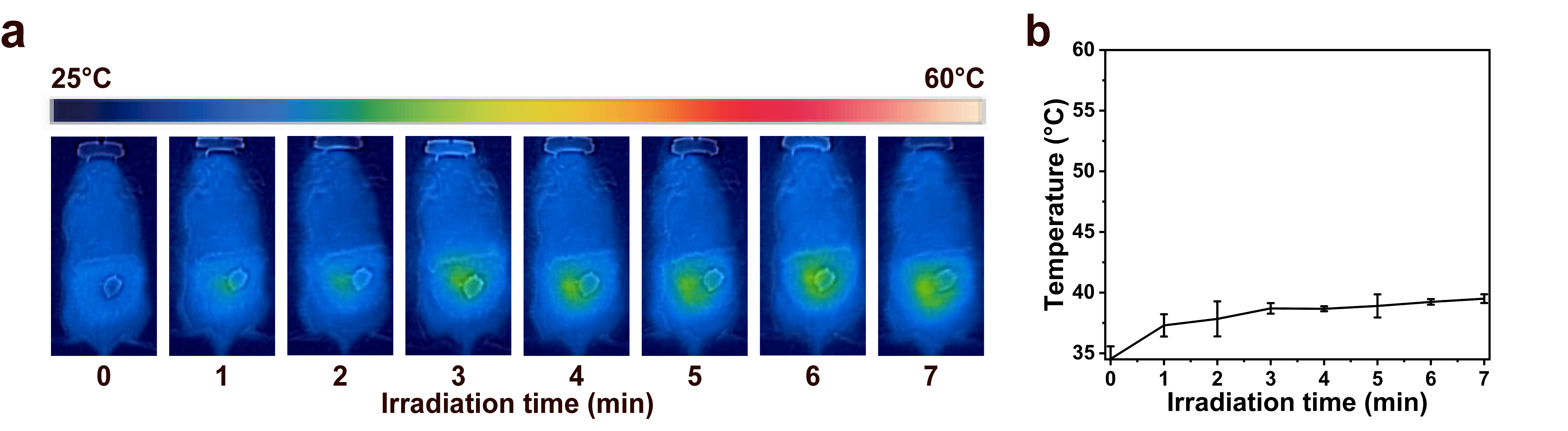


Figure S18 (a) Infrared thermal pictures, and (b) the rise in temperature at the wound sites of mice at the designated periods under 808 nm NIR illumination (2.3 W/cm^2^) with treatments of CeO_2_+TMB (CeO_2_: 30 µg/mL, TMB: 1 mM). Error bars represent the standard deviation from the mean (n = 3).


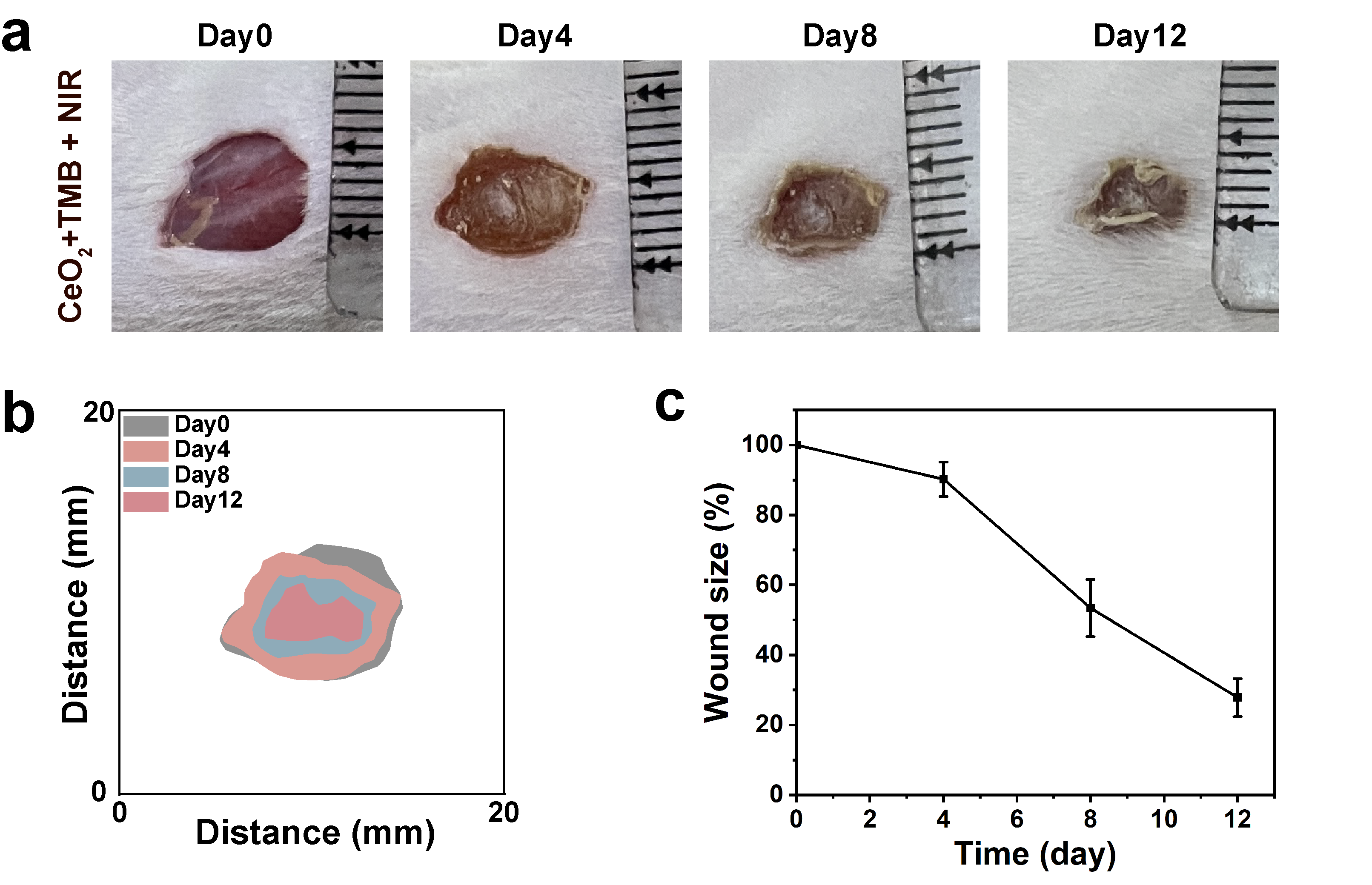


Figure S19 (a) Pictures depicting the progression of wound contraction from day 0 to day 12 in response to the treatments of CeO_2_+TMB + NIR group (808 nm, 2.3 W/cm^2^, 7 min). (b) The chart of the dynamic process of wound healing in CeO_2_+TMB + NIR group within 12 days. (c) Quantitative analysis of the wound size in CeO_2_+TMB + NIR group. Error bars represent the standard deviation from the mean (n = 3).


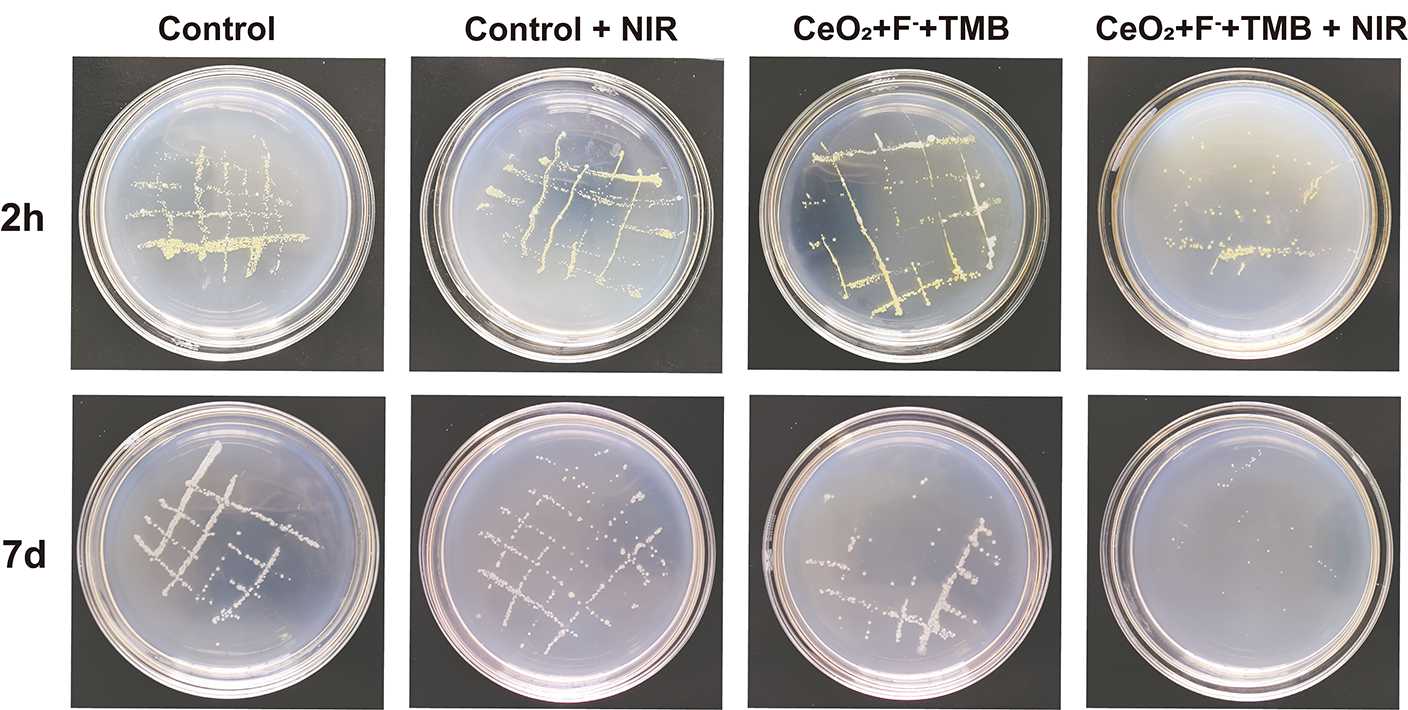


Figure S20 The bacteria were separated from the wound tissue at different intervals and cultured on agar plates.


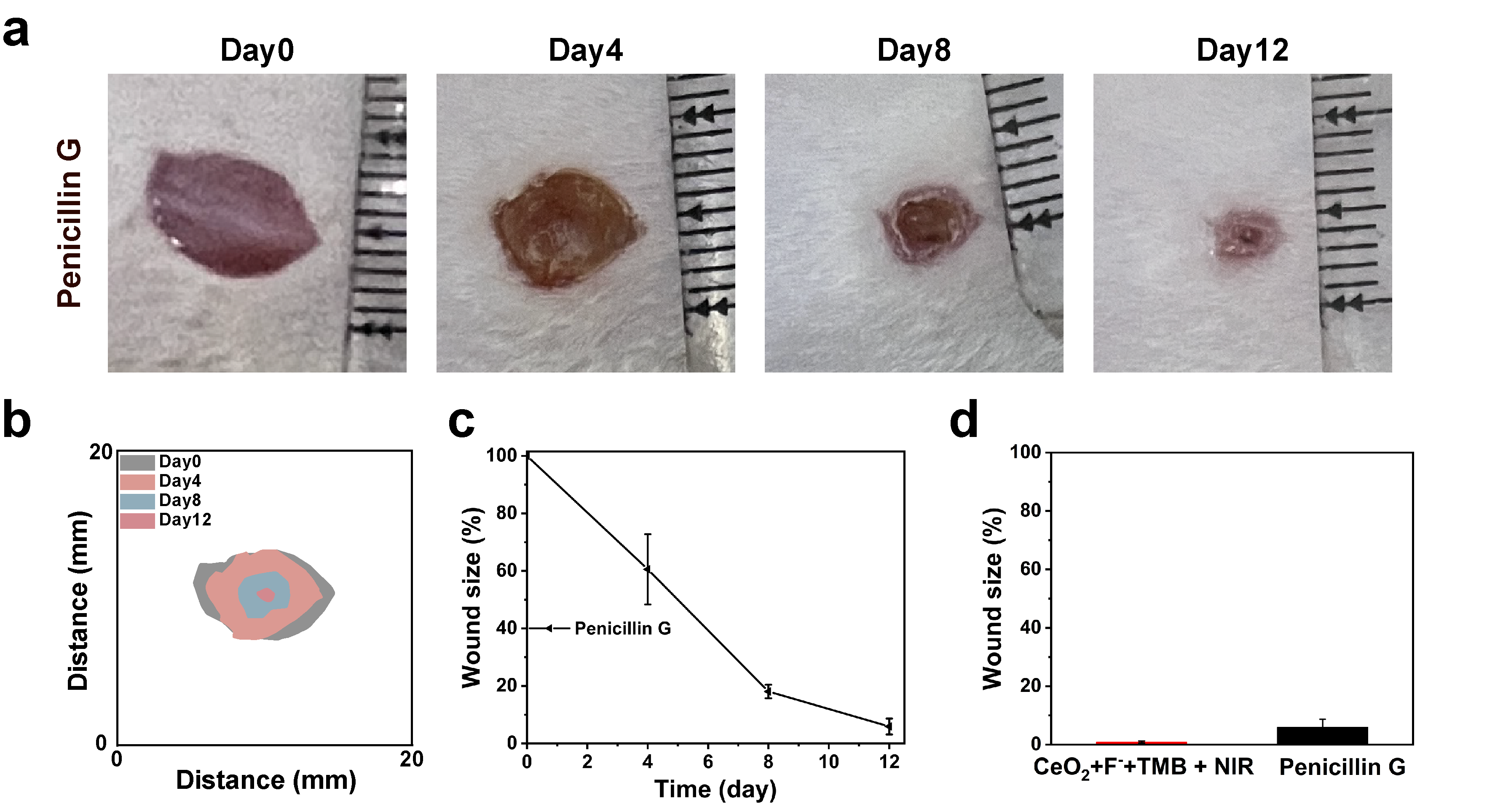


Figure S21 (a) Pictures depicting the progression of wound contraction from day 0 to day 12 in response to penicillin G. (b) The chart of the dynamic process of wound healing within 12 days. (c) Quantitative analysis of the wound size within 12 days. (d) Comparison of wound size on day 12 with treatments of the penicillin G and CeO_2_+F^-^+TMB group. Error bars represent the standard deviation from the mean (n = 3).


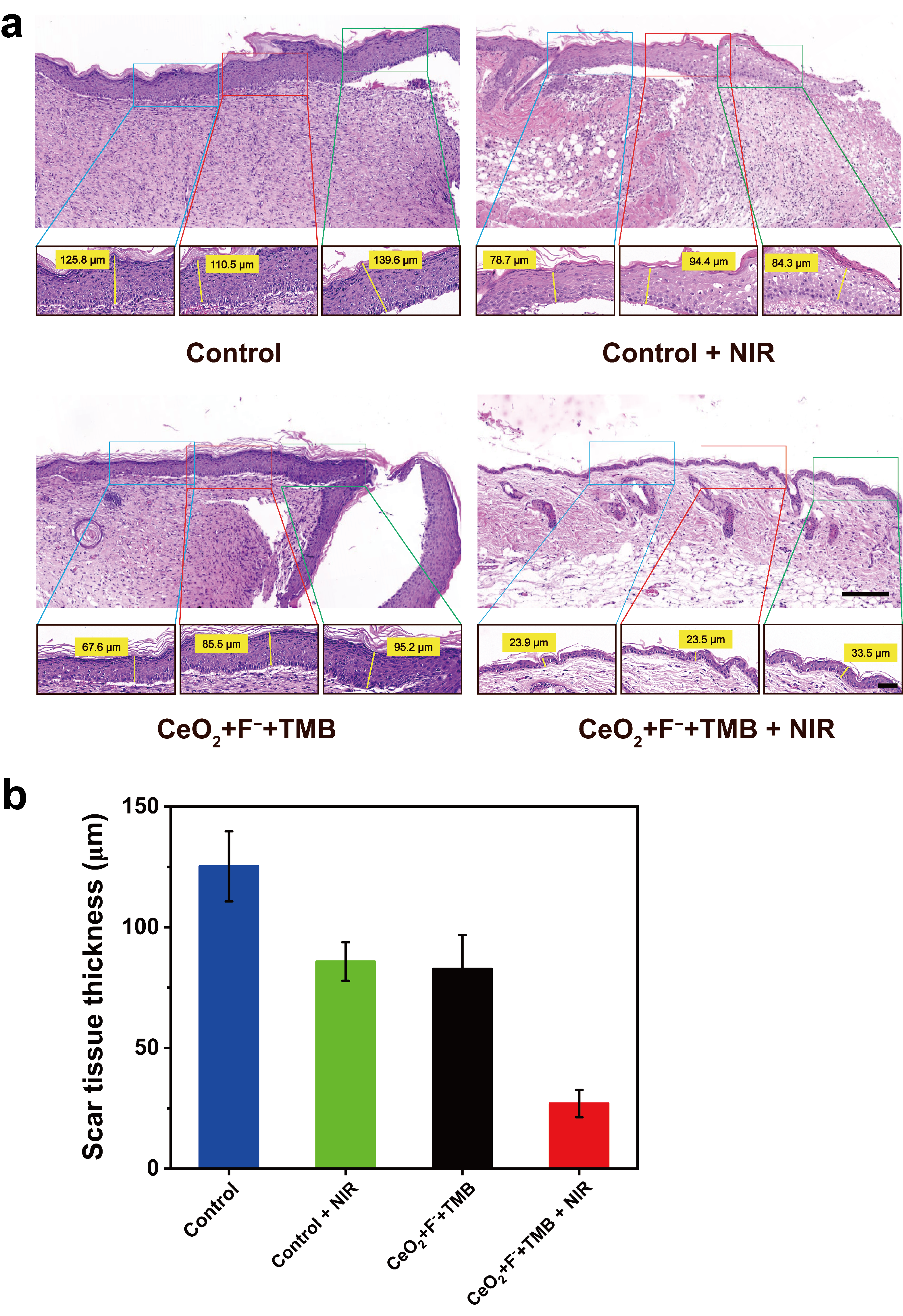


Figure S22 (a) H&E staining images of scar tissue thickness (Scale bar: the top of 200 µm and the bottom of 50 µm). (b) Quantification of scar tissue thickness. Error bars represent the standard deviation from the mean (n = 3).


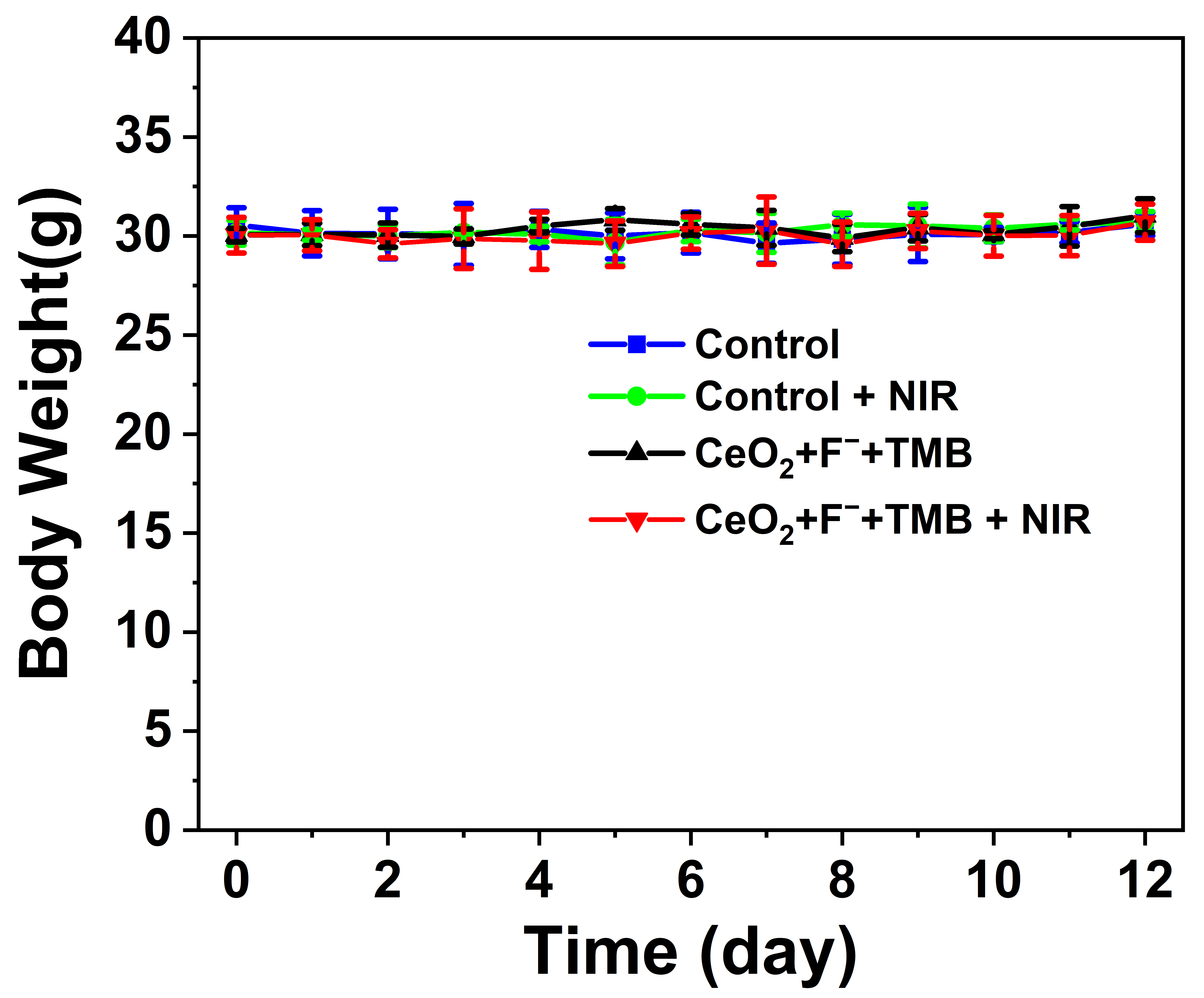


Figure S23 Body weights of different mice groups following treatment. All data are presented as mean ± SD (n =3). Concentration: CeO_2_ (30 µg/mL), F^-^ (400 µM), TMB (1 mM).

**REFERENCES**

[1] F. Muhammad, A. Wang, W. Qi, S. Zhang, G. Zhu, Intracellular Antioxidants Dissolve Man-Made Antioxidant Nanoparticles: Using Redox Vulnerability of Nanoceria to Develop a Responsive Drug Delivery System, ACS Appl. Mater. Interfaces 6 (2014) 19424-19433.

[2] X. Hou, H. Zeng, X. Chi, X. Hu, Pathogen Receptor Membrane-Coating Facet Structures Boost Nanomaterial Immune Escape and Antibacterial Performance, Nano Lett. 21 (2021) 9966-9975.

[3] X. Xie, R. Wang, X. Zhang, Y. Ren, T. Du, Y. Ni, H. Yan, L. Zhang, J. Sun, W. Zhang, J. Wang, A photothermal and self-induced Fenton dual-modal antibacterial platform for synergistic enhanced bacterial elimination, Applied Catalysis B: Environmental 295 (2021) 120315.
